# In silico design of a polypeptide as a vaccine candidate against ascariasis

**DOI:** 10.1038/s41598-023-30445-x

**Published:** 2023-03-02

**Authors:** Francisco M. D. Evangelista, Arnoud H. M. van Vliet, Scott P. Lawton, Martha Betson

**Affiliations:** 1 Department of Comparative Biomedical Sciences, School of Veterinary Medicine, Faculty of Health and Medical Sciences, University of Surrey, Guildford, GU2 7AL UK; 2 Centre for Epidemiology and Planetary Health, Department of Veterinary and Animal Sciences, Northern Faculty, Scotland’s Rural University College (SRUC), An Lòchran, Inverness, IV2 5NA UK

**Keywords:** Protein design, Protein structure predictions, Peptide vaccines, Parasitic infection, Parasitic infection

## Abstract

Ascariasis is the most prevalent zoonotic helminthic disease worldwide, and is responsible for nutritional deficiencies, particularly hindering the physical and neurological development of children. The appearance of anthelmintic resistance in *Ascaris* is a risk for the target of eliminating ascariasis as a public health problem by 2030 set by the World Health Organisation. The development of a vaccine could be key to achieving this target. Here we have applied an in silico approach to design a multi-epitope polypeptide that contains T-cell and B-cell epitopes of reported novel potential vaccination targets, alongside epitopes from established vaccination candidates. An artificial toll-like receptor-4 (TLR4) adjuvant (RS09) was added to improve immunogenicity. The constructed peptide was found to be non-allergic, non-toxic, with adequate antigenic and physicochemical characteristics, such as solubility and potential expression in *Escherichia coli*. A tertiary structure of the polypeptide was used to predict the presence of discontinuous B-cell epitopes and to confirm the molecular binding stability with TLR2 and TLR4 molecules. Immune simulations predicted an increase in B-cell and T-cell immune response after injection. This polypeptide can now be validated experimentally and compared to other vaccine candidates to assess its possible impact in human health.

## Introduction

Ascariasis is a disease caused by the soil-transmitted helminth (STH) *Ascaris*. This is the most prevalent STH worldwide with approximately 446 million people infected and is responsible for a loss of 754,000 disability adjusted life years (DALYs) in 2019^1^. Ascariasis is classified as a Neglected Tropical Disease (NTD) and is typically associated with low- and middle-income regions, such as sub-Saharan Africa, South America, South- and South-east Asia^2,3^. Concurrently, *Ascaris* is also one of the most prevalent intestinal nematode species in the domestic swine^4^. Ascariasis is similar in both humans and pigs, having a potential negative effect on growth, cognitive development and nutritional status^5,6^.The two *Ascaris* species, *A. lumbricoides* and *A. suum*, are usually associated with infections in either humans or pigs, respectively. However, there is potential for zoonotic transmission between host species with *A. suum* infections identified in humans and *A. lumbricoides* infections reported in pigs^3,7^. The two *Ascaris* species are also able to develop viable hybrids^5,8^, reinforcing the need to control the parasite in both human and swine populations.

Preventive chemotherapy through Mass Drug Administration (MDA) is the main control method for ascariasis. This approach has been supported with large-scale screening protocols and improvements to water, sanitation and hygiene (WASH)^2,6^. Recently, there have been increasing concerns about the potential development of resistance to anthelmintic treatment in *Ascaris*^9,10^. Even though it has not become widespread, the appearance of resistance has led to a growing interest in other control/therapeutic methods, such as vaccines^11^. At the point of writing, there is no vaccine that has gone through human clinical trials or that has been approved for commercial use in pigs. The development of a vaccine would help achieve the WHO 2030 Roadmap for NTD target of elimination of ascariasis as a public health problem^2^.

Over the last few decades there have been a number of studies aiming to develop vaccines against *Ascaris*^12–18^. The vaccines in these trials were tested mainly in mice and efficacies ranging from 38 to 77% reduction in larvae burden. One of the issues with using mouse models is that *Ascaris* is not able to complete its life cycle from larvae to adult, so it is not possible to assess efficacy against the latter stage. These assays have used either *Ascaris* recombinant proteins^12–14,16,18^, crude extracts^17^ or a subunit chimeric protein^15^. The latter proved to be the most efficient in reducing larval burden, with a 77%^15^ and 66% reduction^19^ when provided by subcutaneous injection or orally, respectively. This protein used a combination of in silico predicted B-cell epitopes from known vaccine targets (As14, As16 and As37). More recently, using a mixed bioinformatics and in vitro methodology for B-cell epitope selection, ASCVac-1 was able to reduce larvae burden by 50%^20^. Nevertheless, these values are still lower than the 94% efficacy with immunisation using UV-attenuated eggs^21^. Such differences led us to believe that there is still room for improvement and that other antigens or epitopes could be included in a multi-epitope vaccine.

The immune response in ascariasis is still not completely understood. It is however suggested that a T-helper type 2 (Th2) response with eosinophilia is mainly responsible for promoting resistance and controlling this parasite^22–26^. The Th2 cytokines IL-4 and IL-10 are commonly elevated in humans and animals that have been previously exposed to *Ascaris* and are known to affect the recruitment of eosinophils^24,27,28^. Furthermore, it is acknowledged that toll-like receptor (TLR)-2 and TLR-4 are important mediators in the adaptive immune response in helminth infections with cytokines and chemokines release^24,29,30^. Animals without these receptors were found to be more prone to *Ascaris* reinfection due to lower eosinophil levels^24,30^. It is also important to reinforce that antibodies, such as IgA, IgE and IgG, are also relevant in controlling *Ascaris*^12,14,15,17,18,20,24,31–34^. Nevertheless, treatment and prevention of ascariasis requires a balance between the continuous local exposure to IgE and subsequent eosinophilia due to exacerbation of Loeffler syndrome, restrictive lung disease and gastrointestinal symptoms^35–38^.

The aim of this study was to design and test an ascariasis vaccine using an in silico methodology. This approach has already used to develop vaccines for different pathogens including SARS CoV-2^39^, *Mycobacerium tuberculosis*^40^, *Schistosoma mansoni*^41^ and *Onchocerca volvulus*^42^. Here we have used antigens present in *A. lumbricoides* and *A. suum* to devise a multi-epitope vaccine that could prevent infection in multiple hosts and interfere with zoonotic transfer of *Ascaris*.

## Methods

### Selection and retrieval of vaccination targets

**Figure 1:**
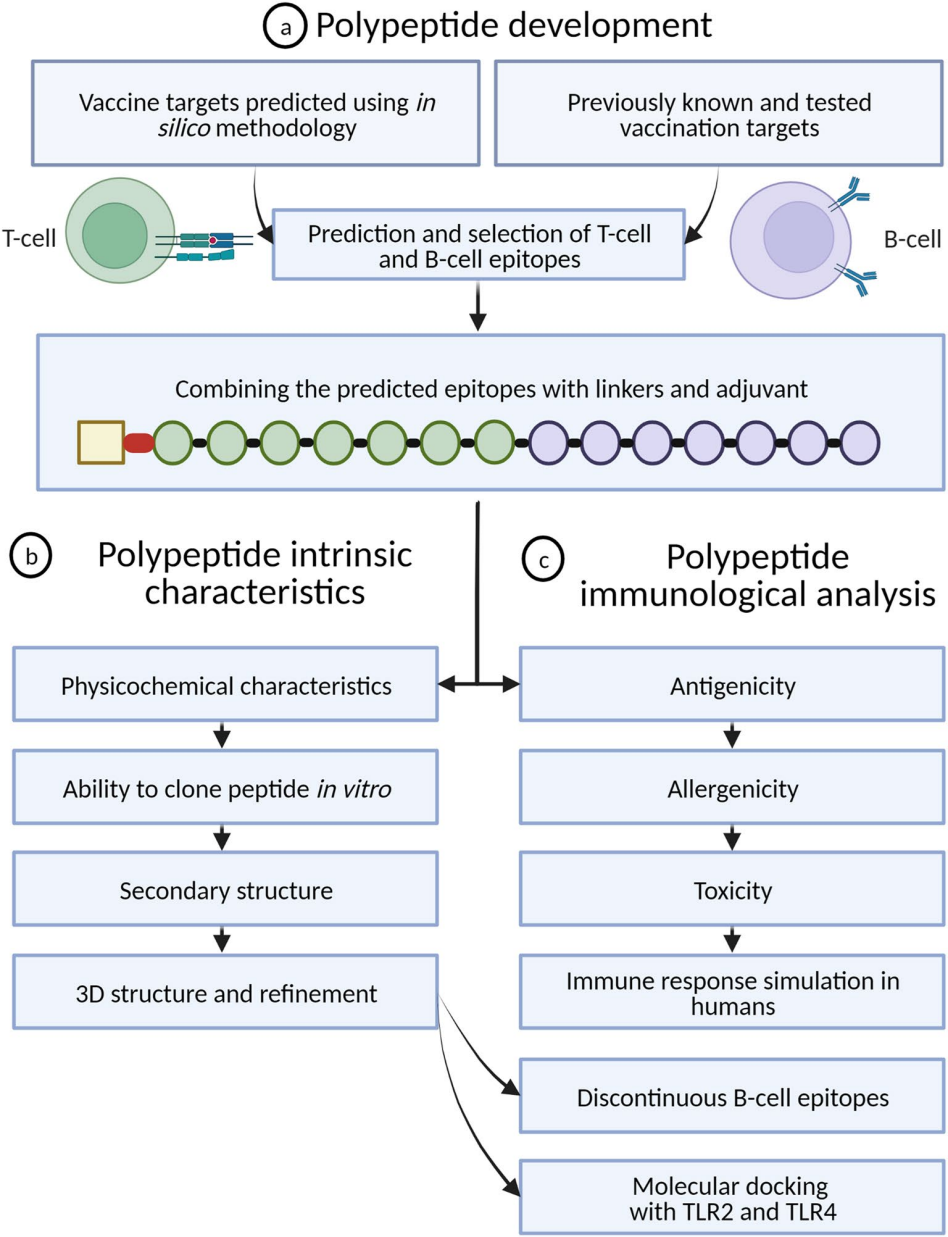
Summary workflow of the approach used to design and analyse the multi-epitope polypeptide. (**a**) Polypeptide development steps, including selection of vaccine targets and respective T-cell and B-cell epitopes. (**b**) Steps of the polypeptide intrinsic characteristics analysis. (**c**) Components of the immunological analysis.

The vaccination targets were selected based on the results and predictions of previous studies. A total of 7 different genes and respective proteins were selected: AgB13X_g094 (ATtype), AgR007_g063 (Apiezo), AgR007_g282 (Altype), AgB13X_g096 (Aproto)^43^, AgB02_g183 (As37), AgR006_g148 (As16), and AgR006_g147 (As14)^12,14,15,18^. The proteins Attype, Apiezo, Altype and Aproto were selected based on a reverse vaccinology framework^43^, while the proteins As37, As16 and As14 are vaccine targets that have been successfully used in vaccination assays^12,14,15,18^. The amino acid (aa) sequences were retrieved from the most recent *Ascaris* proteome (BioProject PRJNA62057) available in WormBase Parasite database^44^. A workflow summary is available in Fig. 1.

### Prediction and selection of helper T-cell epitopes and linear B-cell epitopes

Helper T-cell (HTC) epitopes for the Attype, Apiezo, Altype and Aproto proteins were retrieved from a previous study^43^. HTC epitope predictions for the proteins As37, As16 and As14 were made using the IEDB MHC-II binding predictor v2.22.3 (MHCII-IEDB, available at http://tools.iedb.org/mhcii/). The IEDB-recommended 2.22 prediction Consensus method was used, encompassing the NN-align, SMM-align, CombLib and Sturniolo methods, or NetMHCIIpan^45^. The peptide epitope length of 15 aa with a 9 aa core was selected and predictions were made against the 27 human leukocyte antigen (HLA) allele reference set^46^. For each protein, we selected two non-redundant epitopes (without overlapping aa) that scored between 0 and 2 and were predicted to bind to the largest combined number of unique MHC-II alleles in MHCII-IEDB tool analysis. The selected epitopes were submitted to IL4pred (https://webs.iiitd.edu.in/raghava/il4pred/index.php)^47^ and IL-10Pred (https://webs.iiitd.edu.in/raghava/il10pred/)^48^ to assess their capacity to induce IL-4 and IL-10 production. In IL4pred we used the hybrid-based method in the Virtual Screening module with 0.2 SVM threshold. For IL-10 prediction we used the predict module with a threshold of -0.3 for the SVM based model.

B-cell epitopes for Attype, Apiezo, Altype and Aproto proteins were identified in our previous study^43^. B-cell epitopes in As37, As16 and As14 were identified using Bepipred v2.0 (http://www.cbs.dtu.dk/services/BepiPred/)^49^. The two highest scoring B-cell epitopes with a length between 8 and 40 aa were selected for each protein. These epitopes were then submitted to AbCPE webserver (http://bioinfo.unipune.ac.in/AbCPE/Home.html)^50^ to predict which class of antibody they were capable of inducing.

### Allergenicity, toxicity and epitope identity assessment

The retrieved HTC and B-cell epitopes were evaluated for allergenicity using Allertop 2.0 (https://www.ddg-pharmfac.net/AllerTOP/index.html)^51^ and toxicity with the ToxinPred web server (https://webs.iiitd.edu.in/raghava/toxinpred/index.html)^52^. In ToxinPred, a 10 amino acid peptide fragment length was chosen, and toxicity was predicted using the SVM (Swiss-Prot) and Motif based methods, with 0.0 threshold and 10 e-value cut-offs respectively. Furthermore, epitopes were assessed for identity in both humans and pigs. These epitopes were submitted to a BLASTp (Basic Local Alignment Search Tool protein) search (available at https://blast.ncbi.nlm.nih.gov/Blast.cgi). This was cross-checked by submitting the epitopes to the Multiple Peptide Match tool available in PIR (https://research.bioinformatics.udel.edu/peptidematch/batchpeptidematch.jsp)^53^. Epitopes were matched against both human and pig proteomes using the UniProt/SwissProt option and including the different isoforms. Epitopes that were predicted to be toxic or were found in either humans or pigs were rejected, and a new epitope was selected for testing.

### Design of a multi-epitope vaccine and immunological analysis

The epitopes identified in the previous section were fused together with flexible linkers and a TLR-4 agonist. The flexible linker GSGSG was selected to bind both HTC and B-cell epitopes^15,54^. The synthetic TLR-4 adjuvant RS09 (Sequence: APPHALS) was added to the N-terminal of the vaccine construct using an EAAAK linker^54,55^. The multi-epitope vaccine amino acid sequence was analysed for its immunological properties. Both AntigenPro (http://scratch.proteomics.ics.uci.edu/)^56^ and VaxiJen v2.0 (http://www.ddg-pharmfac.net/vaxijen/VaxiJen/VaxiJen.html)^57^ were utilised to assess the antigenicity of the multi-epitope vaccine construct. The VaxiJen v2.0 server was used with bacteria and parasite models, and a 0.7 threshold was used in both. Allergenicity was predicted using Allertop v2.0 and AllergenFP v1.0 (http://www.ddg-pharmfac.net/AllergenFP/index.html)^58^. Vaccine toxicity assessment was made using the previously mentioned methodology with the ToxinPred webserver. The multi-epitope vaccine is expected to be antigenic, non-allergic and non-toxic. Furthermore, the coverage in the world population was assessed with the IEDB Population Coverage tool (http://tools.iedb.org/population/)^59^. This was done by selecting “World” as target population, calculating MHC Class II separately and restricting the MHC alleles to the previously used 27 HLA reference set.

### Immune simulation scenario in humans

The C-IMMSIM web server (https://kraken.iac.rm.cnr.it/C-IMMSIM/)^60^ was utilized for in silico simulations of human immune response to the multi-epitope vaccine. We considered two immunization schedules: one with two doses with a 4-week interval and a second scenario with a third dosage at the 8th week. In C-IMMSIM eight hours in real life correspond to one simulation step. Therefore, the simulations were conducted with two injections at the time step 1 and 84 (vaccine injection happens during the first simulation step), with a third injection at step 168 for the second scenario. Both scenarios accounted for a total of 1096 simulation steps (one year). The injections in the tool were selected as vaccines with no LPS (as this is not present in the tested vaccine), random seed = 12,345, and the host HLA selection was modified to include the MHC-II alleles DRB1*07:01 and DRB1*15:01, as both are part of the HLA allele reference set and are known to react distinctly to different *Ascaris* antigens^46,61^.

### Prediction of physicochemical characteristics and secondary structure

The ProtParam web server (http://web.expasy.org/protparam/)^62^ was used to evaluate the physicochemical characteristics of the selected multi-epitope vaccine. The analysed characteristics included molecular weight, in vitro and in vivo half-life, instability index, theoretical isoelectric point (pI), aliphatic index and grand average of hydropathicity (GRAVY). The vaccine solubility upon overexpression in *Escherichia coli* was assessed with SOLPro (http://scratch.proteomics.ics.uci.edu/)^63^. The vaccine secondary structure (2D) was predicted with the PSIPRED 4.0 (http://bioinf.cs.ucl.ac.uk/psipred/)^64^ and RaptorX Property Prediction (http://raptorx.uchicago.edu/StructurePropertyPred/predict/)^65^ tools. These programs were utilised to evaluate the secondary structure of the vaccine regarding the presence of coils, α-helixes, β-sheets and disordered domains with ~ 84% accuracy^64,65^.

### In-silico optimization and cloning of designed multi-epitope vaccine

The reverse translation and codon optimization of the multi-epitope vaccine were performed with the Java Codon Adaptation Tool (JCat) webserver (http://www.jcat.de/)^66^ to prepare for future experimental work. This codon optimization was performed for expression in an *Escherichia coli* K12 derivative, while outputs of the Codon Adaptation Index (CAI) and the percentage of GC content were used to evaluate this step. The CAI provides an indication of how likely it is that an organism can express a heterologous gene. A good optimization has a CAI above 0.80 and a GC content between 30% and 70%, respectively. The NdeI and XhoI restriction sites were added to the N and C-terminus of the optimized nucleotide sequence, respectively. The final sequence was inserted into the pET30a (+) vector and tested for its predicted viability using the SnapGene software (from Insightful Science; available at https://snapgene.com).

### Tertiary structure prediction, refinement, and validation

The three-dimensional (3D) model of the multi-epitope protein was created using the RaptorX Structure Prediction tool (http://raptorx.uchicago.edu/ContactMap/)^67^. The 3D structures are predicted using a de novo deep-learning model that predicts a contact map based on a multiple sequence alignment from the primary sequence and is especially effective with proteins that do not possess various sequence homologues. The first-ranked balanced-energy protein model was retrieved and refined using GalaxyRefine web server (https://galaxy.seoklab.org/cgi-bin/submit.cgi?type=REFINE)^68^. These tools were amongst the highest rated tools for protein 3D modelling and refinement in the CASP13 assessments^69,70^. Both the unrefined and refined 3D models were validated using the ProSA-Web web server (https://prosa.services.came.sbg.ac.at/prosa.php)^71^ and the PROCHECK^72^, ERRAT^73^, VERIFY 3D^74^ and PROVE^75^ programs available in SAVES v6.0 (https://saves.mbi.ucla.edu/). ProSA-Web analyses the protein 3D model by calculating an overall quality Z-score and comparing it to the score of protein structures obtained through X-ray analysis, NMR spectroscopy and theoretical calculations. The PROCHECK program validates a protein structure by using a Ramachandran plot and visualizing the percentage of aa residues that are in favoured, allowed and disallowed regions. While ERRAT is used to assess the overall model quality at a 0 to 100 scale using atomic composition, VERIFY 3D checks the model quality from the 3D model and compares it directly to its amino acid composition. Finally, PROVE assesses model quality by using an atom volume-based validation.

### Discontinuous B-cell epitope prediction

Besides the linear B-cell epitopes already described, there was the need to assess the discontinuous B-cell epitopes as they are estimated to account for over 90% of the B-cell immunogenic response^76^. The presence of discontinuous B-cell epitopes formed from protein folding was analysed with the ElliPro server (http://tools.iedb.org/ellipro/)^77^. A minimum score of 0.5 and a maximum distance of 6 Angstrom was used and predicted discontinuous and linear epitopes were retrieved.

### Molecular docking of designed vaccine with TLR2 and TLR4

Immune response against *Ascaris* has been reported to be supported by both TLR2 and TLR4 interactions^24,30^. The ClusPro v2.0 web server (https://cluspro.bu.edu/home.php)^78^ was used to dock the TLR2 (PDB ID: 6NIG)^79^ and TLR4 (PDB ID: 4G8A)^80^ proteins with the multi-epitope vaccine, individually. ClusPro protein docking was used with the vaccine as ligand and TLR2 and TLR4 as receptors. The highest ranked model for each docking prediction was retrieved and evaluated with PRODIGY (https://wenmr.science.uu.nl/prodigy/)^81^. PRODIGY was used to assess the binding affinity, dissociation constant, and the number of contacts created between the multi-epitope vaccine and both TLR2 and TLR4 at 37 °C (protein–protein complexes). The number and type of contacts was also predicted using the LigPlot + v2.2.7 DIMPLOT option^82^. The iMODS web server (http://imods.chaconlab.org/)^83^ was used for a fast molecular dynamics study to define and calculate the protein flexibility following molecular docking. The Basic interface was used with the CA option for the Coarse Grain model representations.

## Results

### Selected vaccination targets and respective epitopes for vaccine design

A total of seven *Ascaris* amino acid protein sequences were retrieved from the WormBase database to design a multi-epitope vaccine against ascariasis. Four proteins highly expressed in adults (ATtype, APiezo, ALtype and Aproto) and three proteins expressed in both larval and adult stages (As37, As16 and As14), were selected based on recent bioinformatics analysis^43^ and previous immunization trials^12,14,15,18^. According to transcriptomic and immunolocalization data, these proteins are mostly located in the muscle (APiezo and ALtype), muscle and hypodermis (As37), ovaries (AProto and ATtype), or are secreted (As16 and As14)^8,13,14,84^.

As T-helper cell epitopes have already been described for AProto, ATtype, ALtype and APiezo^43^, we selected two T-helper epitopes for As37, As16 and As14 based on the results of the MHCII-IEDB tool. A total of 14 HTC epitopes with a length of 15 aa were selected, two for each protein. These epitopes were predicted to be non-allergic, non-toxic and not present in both humans and pigs according to BLASTp and PIR (Table 1). Twelve of the fourteen HTC epitopes were predicted to induce IL-4 or IL-10 production (Supplementary Table S1).

**Table 1:** List of proteins and their respective epitopes used in the multi-epitope vaccine.

WormBase protein identifier	Protein name	Helper T-cell epitopes	B-cell epitopes
AgB13X_g094	ATtype	LRLLRALRPLRVINR FKNFGMAFLTLFRIA	DATGVDMQPVENYN SIPPKSVER
AgB13X_g096	AProto	HTFRRFITAISLLDR NQEGVVHILSRKIFD	LSQSDHHILPRFANFVDDRTESLRSVTIQLLCSLR RQQFTLTFPYFSDGKFK
AgR007_g063	APiezo	NCLKYFANFFFYRFG SLFLRPMRVALALLN	LLSVHLKNDDDSIEST VDPSFDPVIPKEEVI
AgR007_g282	ALtype	NNNFHTFPAAILVLF ERSLLCLTLSNPLRK	ALNDETHIHRNNN SNEEDRGPVYNA
AgB02_g183	As37	TTELKQDNRFSFRLD VMVMEFRAKSILKPT	PQGAPTFTRKPQILQKTSDSGD PLDDGADDA
AgR006_g148	As16	AEYEKAHAAAIAKFS YTNKFKAFKAELKAH	EGQTPSRVPPF EDAKLNGIQKRQKIKETME
AgR006_g148	As14	KITSLLQSLPAAVQA KVLIIFVAIVVIAFA	TQMQQGKARAEAA DNPNLKGREKQQKITSL

As before, B-cell epitopes were already selected for some proteins. For As37, As16 and As14, the two highest scoring epitopes in Bepipred 2.0 with a length between 8 and 40 aa, were selected. As before, we retrieved 14 non-allergic, non-toxic B-cell epitopes that were not present in both humans and pigs (Table 1). All B-cell epitopes were predicted to bind to IgG.

### Multi-epitope vaccine is predicted to have the intended immunological characteristics

**Figure 2:**
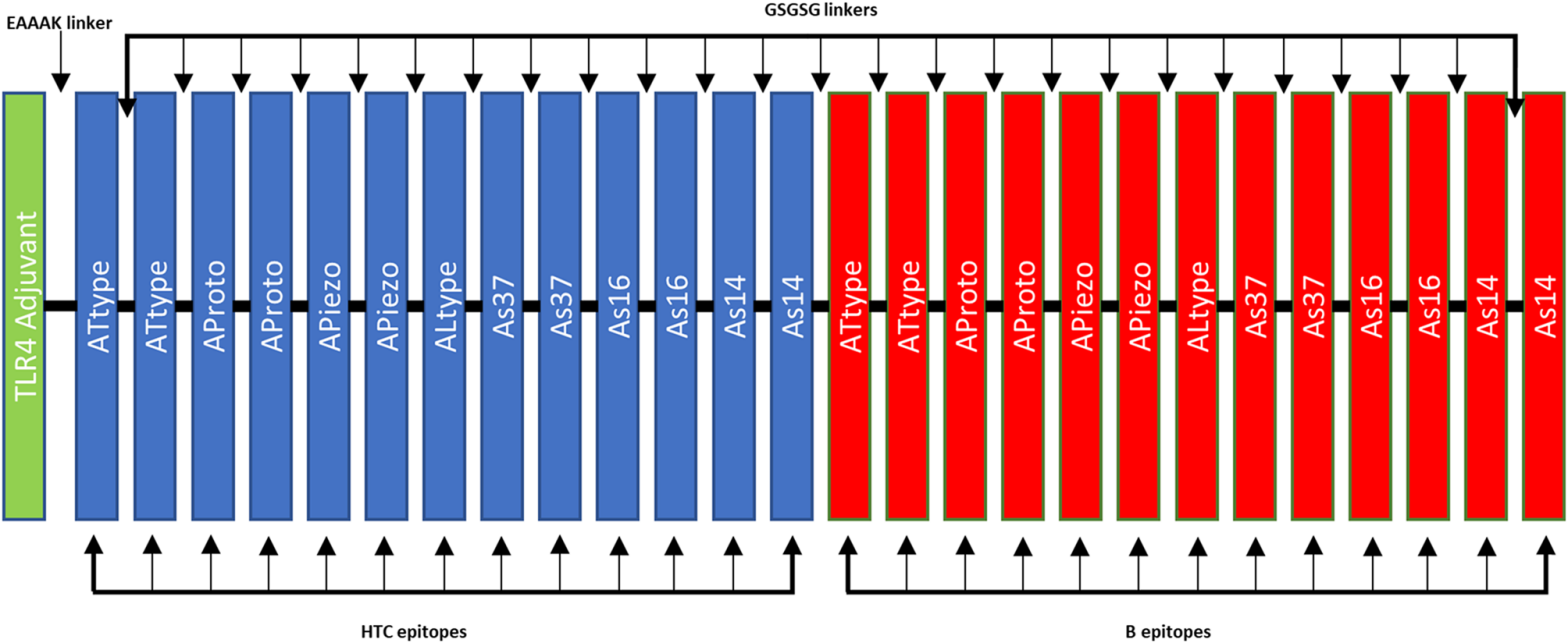
Schematic representation of the final multi epitope vaccine. The sequence contains 579 amino acids with the RS09 TLR4 adjuvant (green) linked at the N-terminal through an EAAAK linker. Both Helper T-cell (blue) epitopes and B-cell epitopes (red) are linked with a GSGSG linker.

The selected 14 T-helper epitopes were linked together with the 14 B-cell epitopes due to the flexible linker GSGSG. The TLR-4 synthetic adjuvant RS09 was included in the N-terminal of the vaccine, before HTC epitopes, thanks to an EAAAK linker^54,55^. The final vaccine has 579 amino acids, and a schematic representation of its design is available in Fig. 2. The vaccine protein sequence can be seen in Supplementary Data S1.

The multi-epitope vaccine was predicted to be antigenic in both VaxiJen 2.0, bacteria and parasite models, and AntigenPro, with scores of 1.1752, 0.8079, and 0.729925, respectively. Both AllergenFP and Allertop 2.0 predicted the vaccine to be non-allergenic while the ToxinPred webserver found it to be non-toxic. Furthermore, the vaccine was calculated to be able to cover 99.98% of the world population.

### Immune simulation highlights an immunological response consistent with actual vaccination outcomes

**Figure 3:**
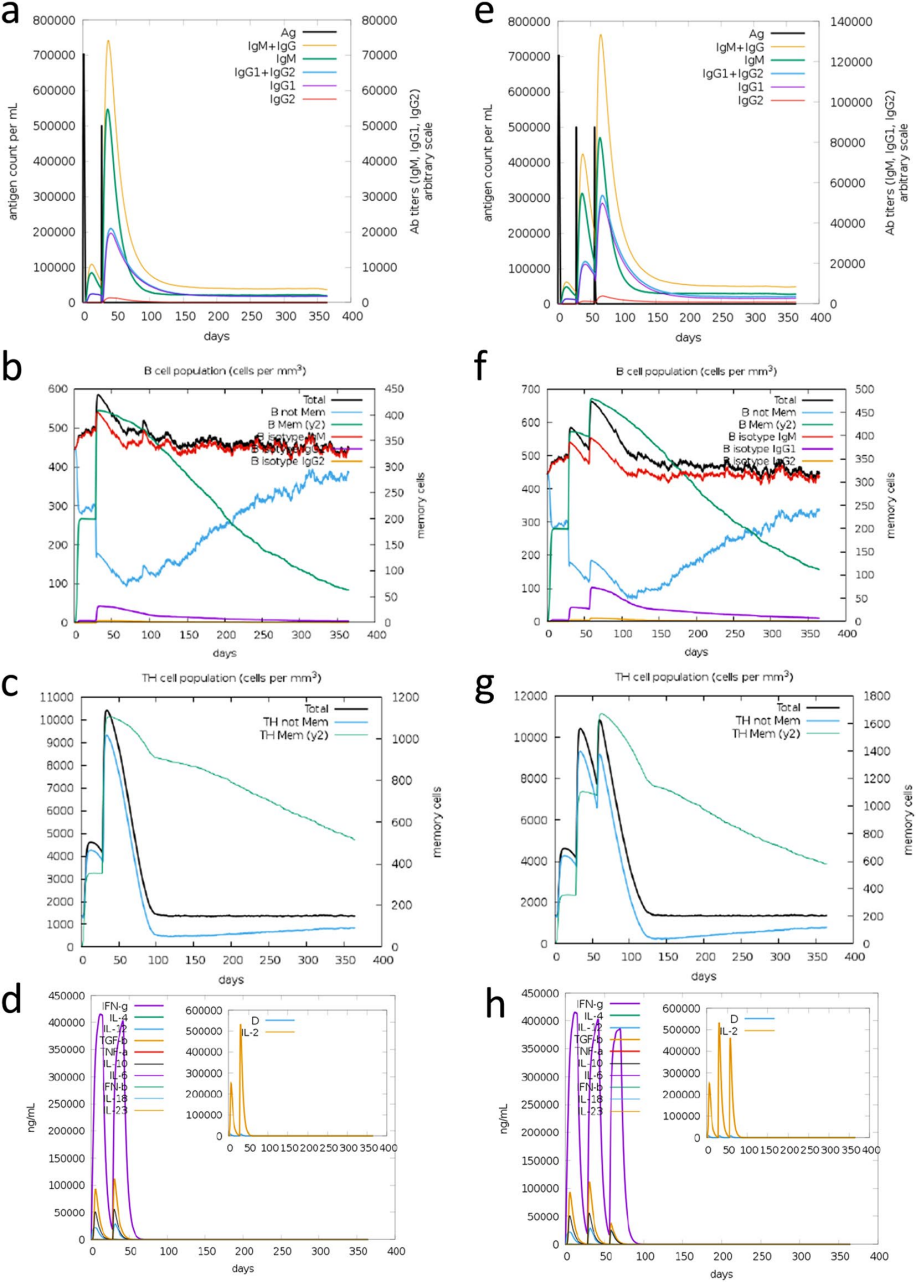
C-ImmSim in silico immune simulation with the designed vaccine complex. (**a–d**) Immune simulation with one booster after four weeks. (**e–h**) Immune simulation with two boosters at four and eight weeks. (**a**, **e**) Antibody titer following vaccine injection. (**b**, **f**) B-cell population upon vaccine injection. (**c**, **g**) The evolution of T-helper cell population. (**d**, **h**) Cytokines and interleukins concentration.

The immune simulation scenarios predicted by the C-ImmSim server predicted similar behaviours for the two different vaccination schedules (Fig. 3). Both scenarios were characterized by a secondary immune response with a rapid increase in the populations of B-cell and HTC, stimulating the high levels of IgM and IgG in response to vaccine injection. Following each injection, the levels of IgG increased proportionally higher than IgM, which further reinforces the characterization as a secondary immune response. Both memory B-cells and HTCs were especially elevated, suggesting long-lasting immune responses. When comparing both scenarios, the three-dose scenario had a significantly higher antibody titer for both IgM and IgG and a slightly higher memory B-cell count after one year. The total level of HTC, specifically memory HTC, was similar between the two injection schemes, both after injections and one year after. The levels of both IFN-γ and IL-2 were similar, independently to the number of boosters used.

### Physicochemical and secondary structure of the designed polypeptide

**Figure 4:**
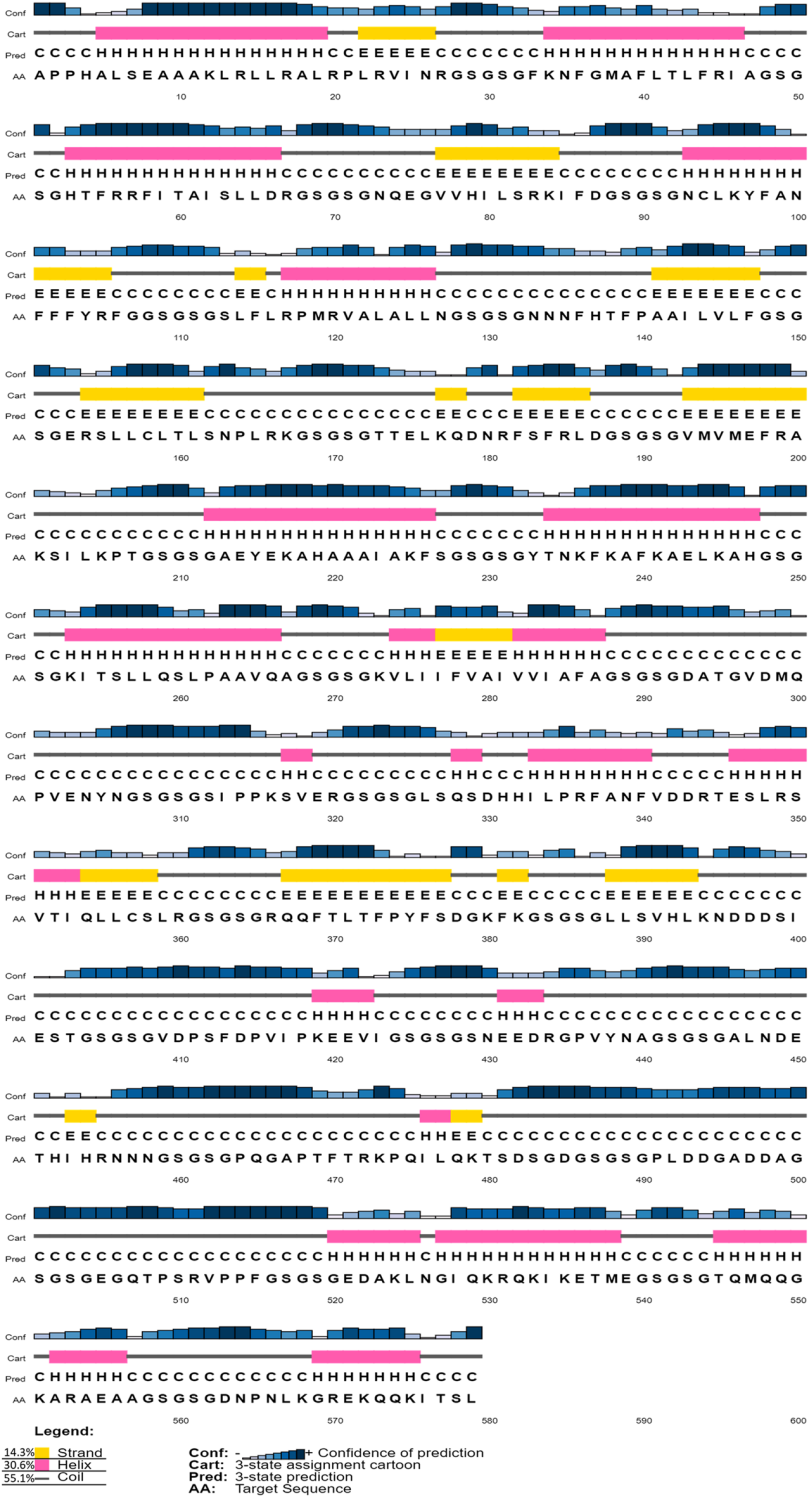
The vaccine secondary structure as estimated by PSIPRED 4.0. The predicted 2D structure contains coils (55.1%), α-sheets (30.6%) and β-strands (14.3%).

**Figure 5:**
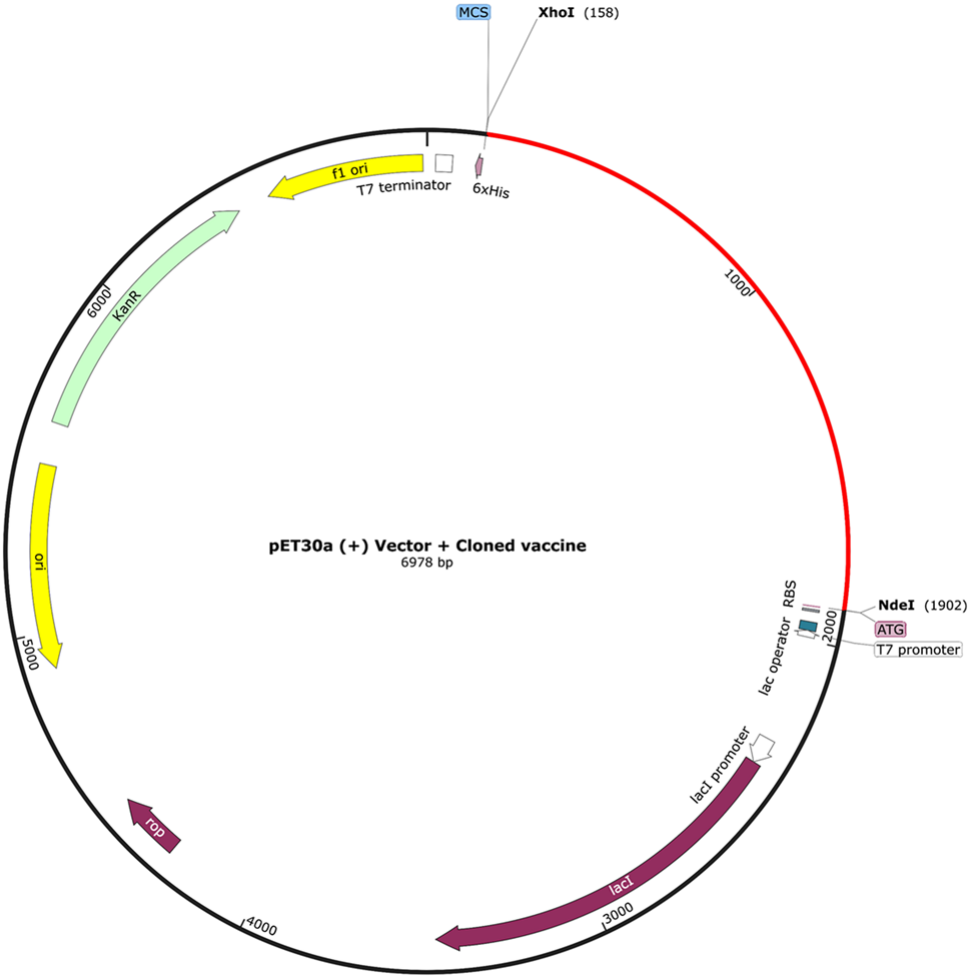
In silico cloning into the pET30a(+) expression vector. The red circle component represents the vaccine coding sequence while the black represents the vector backbone.

The polypeptide molecular weight was predicted to be 59.7 kDa with a pI of 9.58. An instability index of 28.52 indicated the protein stability (proteins with an index lower than 40 are considered stable) while a GRAVY of -0.363 predicts the vaccine as hydrophilic in nature^85^. The estimated aliphatic index of 67.94 points towards the thermostability of the multi-epitope protein^86^. The multi-epitope protein's half-life was predicted as 4.4 h in in vitro mammalian reticulocytes, over 20 h in yeast, and around 10 h in *E. coli*. The final vaccine peptide secondary structure was estimated to be 55.1% coils, 30.6% α-sheets, and 14.3% β-strands (Fig. 4). The disordered domains are predicted to be 50% according to the RaptorX Property Prediction tool. A solubility score of 0.935892, indicating that the vaccine was soluble after overexpression in *E. coli*. The JCat tool was able to generate an optimized DNA sequence for the vaccine sequence to be expressed in *E. coli* (strain k12). The obtained codon sequence has 1737 nucleotides with a predicted GC content of 51.58% and a CAI of 1, optimal for protein expression. The DNA sequence content is available in Supplementary Data S2 and S3. The optimized codon sequence was successfully inserted into the pET30a (+) expression vector using SnapGene (Fig. 5). Both solubility scores and successful sequence insertion reinforce the likelihood of good protein expression when using *E. coli* production model.

### Vaccine 3D model

**Figure 6:**
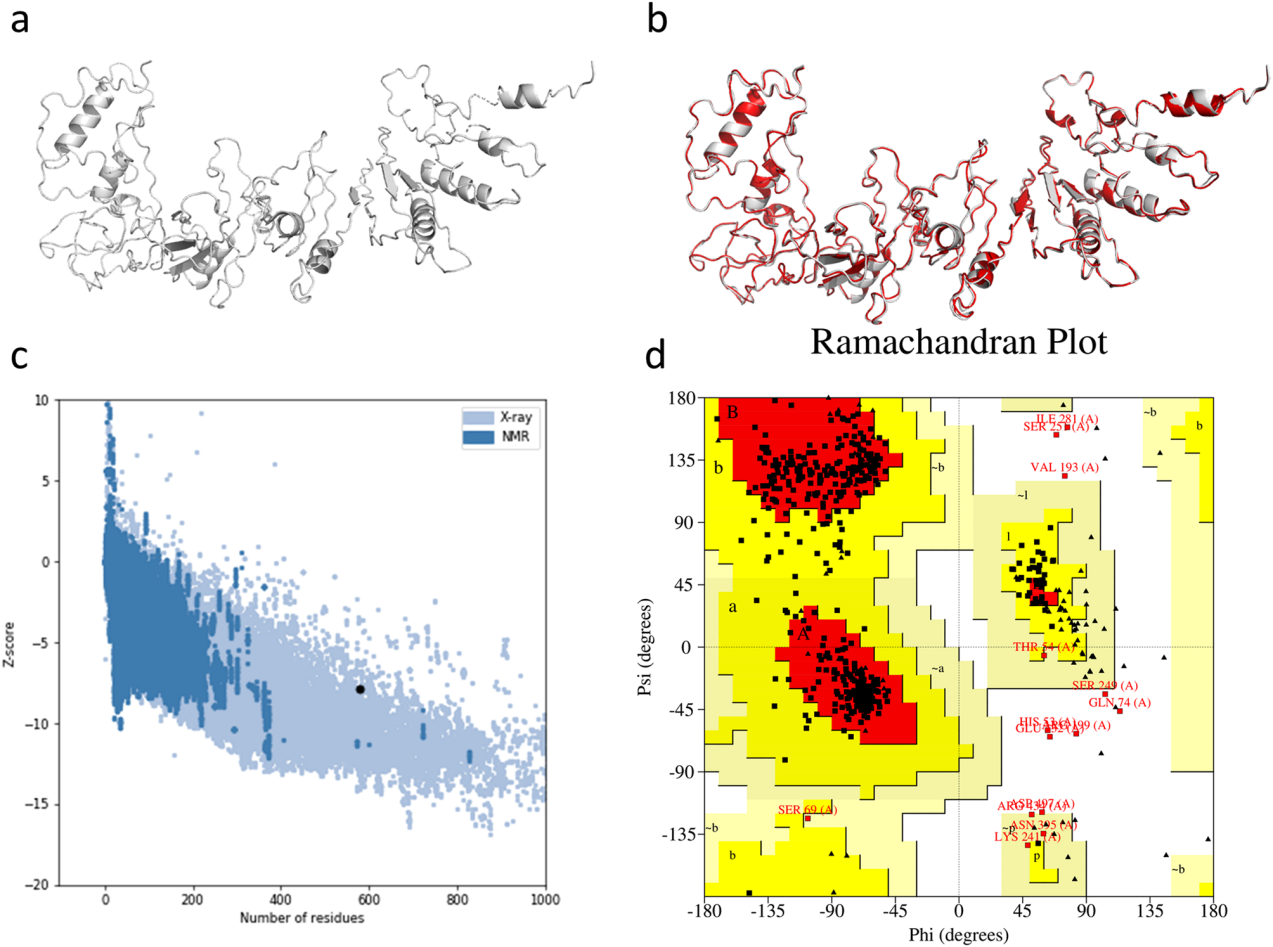
Vaccine 3D modelling, refinement, and assessment. (**a**) The 3D model produced by RaptorX (gray). (**b**) The refined 3D model with GalaxyRefine (red) overlapped with the original RaptorX 3D model (gray). (**c**) The Z-score (-7.9) obtained with ProSa-Web. (**d**) The Procheck Ramachadran plot showing 80.8% of the residues in favoured regions, 17.3% in allowed regions, and 2% in disallowed regions.

The first 3D protein model predicted by RaptorX Structure Prediction also had the lowest RMSD of 13.039 and was selected for refinement (Fig. 6a). When compared to the original model, the first refined model provided by GalaxyRefine had an improved Clash Score from 20.8 to 13.4, a Rama favoured value increase from 86.1 to 92.0, MolProbity decrease from 2.4521 to 2.119, RMSD from 0 to 0.444, GDT-HA from 1 to 0.9404, and an increase in poor rotamers from 0.5 to 0.7 (Fig. 6b). This improved model was validated through a lower Z-score in ProSa-Web, -7.87 to -7.9, and improved residue positions in the Ramachandran plot in Procheck (Fig. 6c,d). The Procheck tool calculated that the proportion of residues in favoured and allowed regions improved from 71.9% and 26.1% to 80.8% and 17.3%, while the residues in disallowed regions remained 2.0%. While ERRAT failed the model with a 76.52% score and PROVE failed the model due to 11.5% of the atoms being buried in an outlier position, VERIFY 3D passed the model with a score of 86.53%. This model was then used in ElliPro’s prediction for discontinuous B-cell epitopes. In this, the polypeptide is expected to have eight discontinuous epitopes that scored between 0.518 and 0.782. These epitopes have a total of 320 amino acids and their size ranged between seven and 115 residues (Supplementary Table S2).

### Designed vaccine is predicted to interact and bind with TLR2 and TLR4

**Figure 7:**
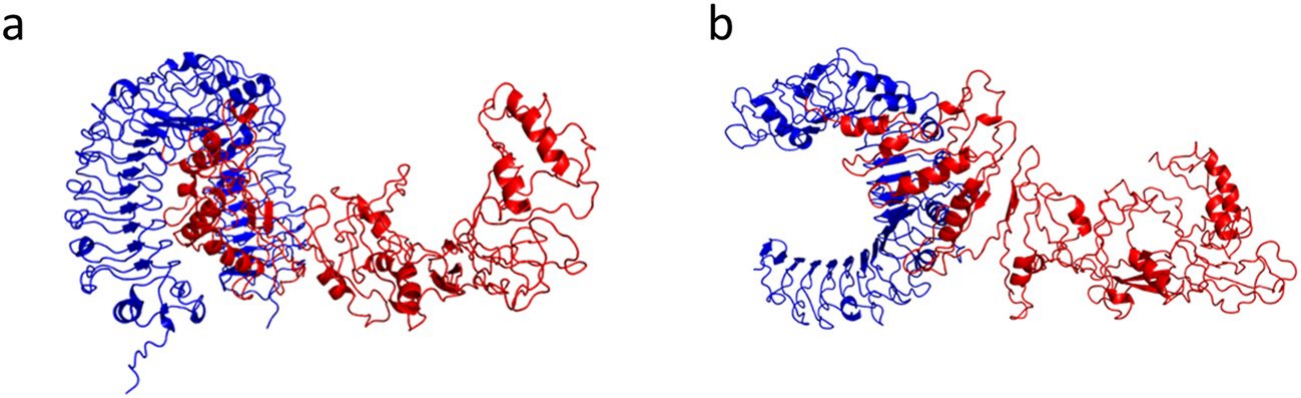
Molecular docking complexes predicted by Cluspro 2.0. (**a**) TLR4 (blue) and vaccine (red). (**b**) TLR2 (blue) and vaccine (red).

**Figure 8:**
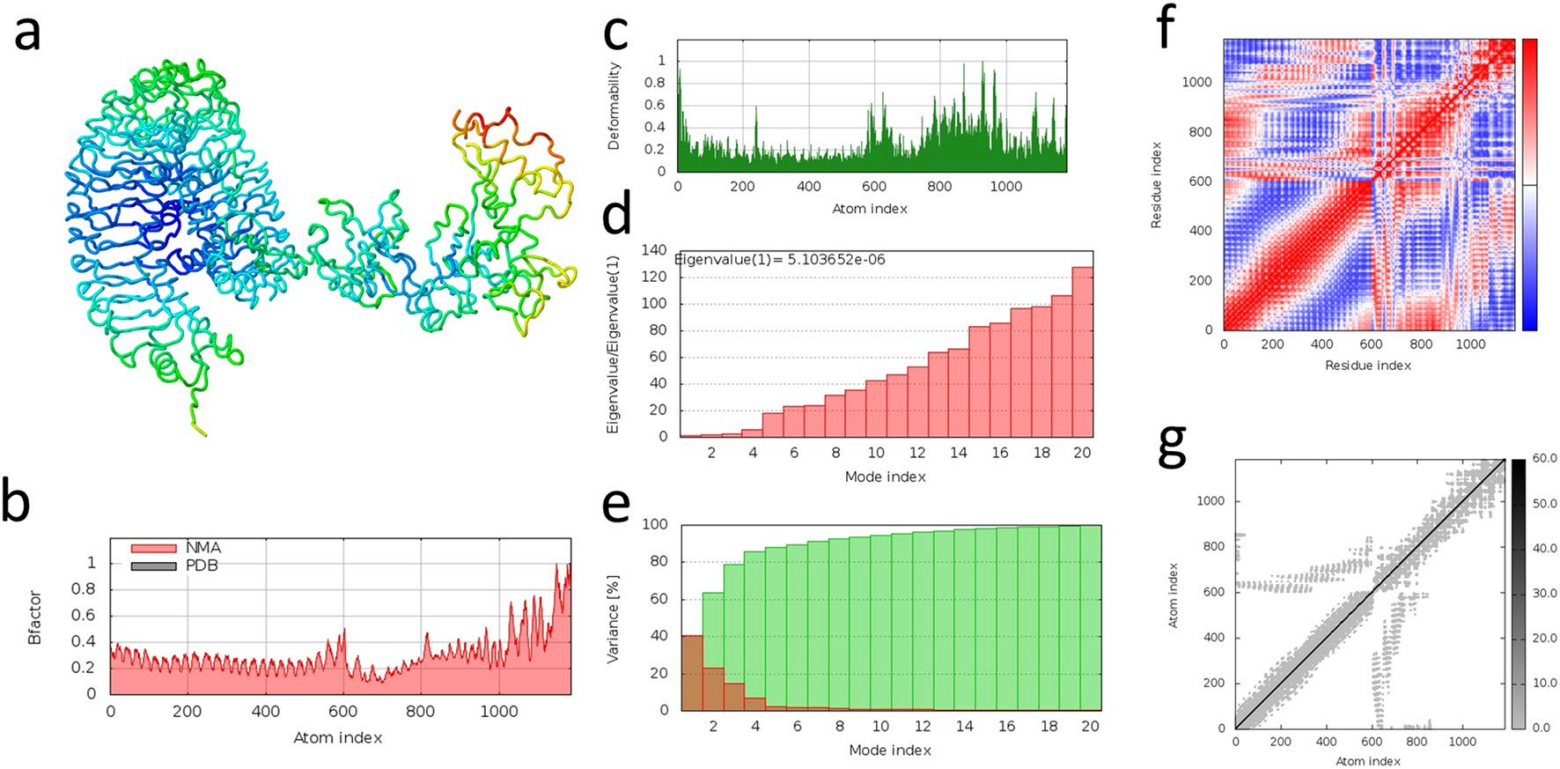
Molecular dynamics analysis of the TLR4 and vaccine complex by the iMODS server (https://imods.iqfr.csic.es/). (**a**) TLR4 and vaccine docking complex Normal Mode Analysis (NMA) highlighting areas of low mobility (blue) to high mobility (red). (**b**) B-factor values with regional mobility scores from 0 (lowest) to 1 (highest). These values are experimentally similar to NMA mobility analysis in a. (**c**) Deformability index of each residue in the complex with higher values reflecting a higher capacity to deform. (**d**) Eigenvalue where the lower it is, the easier it is to deform the complex. (**e**) Variance is inversely related to the eigenvalue. Green indicates cumulative variances while red indicates individual variances. (**f**) Covariance map with correlated (red), uncorrelated (white) or anti-correlated (blue) motions. (**g**) Elastic network where darker grey areas indicate higher stiffness.

**Figure 9:**
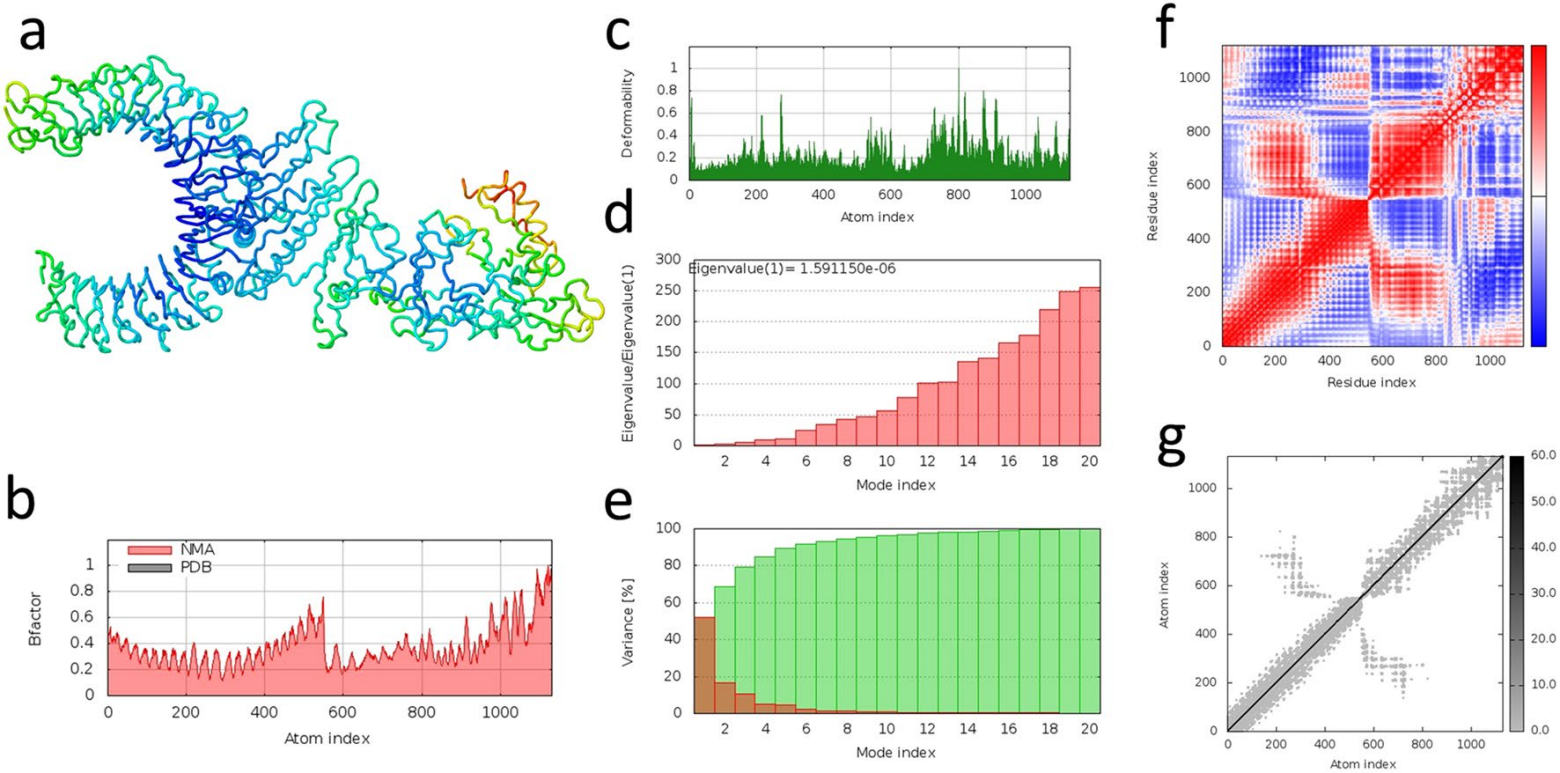
Molecular dynamics analysis of the TLR2 and vaccine complex by the iMODS server (https://imods.iqfr.csic.es/). (**a**) TLR2 and vaccine docking complex Normal Mode Analysis (NMA) highlighting areas of low mobility (blue) to high mobility (red). (**b**) B-factor values with regional mobility scores from 0 (lowest) to 1 (highest). These values are experimentally similar to NMA mobility analysis in a. (**c**) Deformability index of each residue in the complex with higher values reflecting a higher capacity to deform. (**d**) Eigenvalue. The lower the value, the easier it is to deform the complex. (**e**) Variance is inversely related to the eigenvalue. Green indicates cumulative variances while red indicates individual variances. (**f**) Covariance map with correlated (red), uncorrelated (white) or anti-correlated (blue) motions. (**g**) Elastic network where darker grey areas indicate higher stiffness.

We selected the first-ranked complex models between the vaccine and both TLR4 and TLR2, as predicted by ClusPro 2.0 (Fig. 7a,b). The PRODIGY server estimates a strongest binding affinity between the vaccine and TLR4 (− 19.0 kcal/mol) than with TLR2 (− 12.6 kcal/mol), which is supported by a lower dissociation constant (4.20 × 10^–14^ M and 1.30 × 10^–09^ M, respectively) and a higher number of contacts, 177 between the vaccine and TLR4 and 108 with TLR2 (Supplementary Table S3). LigPlot + corroborated the PRODIGY contact results by predicting more hydrogen bonds (22 and 20), non-bonded contacts (212 and 150) and salt bridges (4 and 2) between the vaccine and TLR4 than with TLR2 (Supplementary Tables S4–S9). The iMODS molecular dynamics study results for TLR4 and TLR2 vaccine-binding complexes can be observed in Figs. 8 and 9, respectively. The normal mode analysis (NMA) analysed the complex mobility where the areas highlighted in blue colour represent the areas with the lowest mobility and red the areas with the highest mobility (observed in Figs. 8a and 9a). The B-factor value scores corroborate the NMA analysis (Figs. 8b and 9b). The deformability index graph highlights that the areas in the beginning of the TLR molecules and in the centre of the polypeptide molecule are the ones with the higher capacity to deform (Figs. 8c and 9c). Furthermore, the calculated eigenvalues of 5.103652e-06 and 1.591150e-06 for TLR4 and TLR2 complexes, respectively, indicate a slightly higher stiffness in the TLR4-vaccine complex (Figs. 8d and 9d). The variance graph is inversely related to the eigenvalues and highlights in red and green the individual and cumulative variances, respectively (Figs. 8e and 9e). The covariance map (Figs. 8cf and 9f) and the elastic network (Figs. 8g and 9g) underpin that the areas with correlated motions correspond to the areas with higher stiffness within the docking complexes. These results suggest that both complexes are stable, with the TLR4-vaccine complex requiring higher energy to be deformed and, therefore, aligning with the previous PRODIGY results.

## Discussion

In this work, we have designed a multi-epitope polypeptide as a vaccine component against *Ascaris* using an in silico approach. The protein was predicted to be antigenic, non-allergenic and non-toxic, while also being stable and soluble. Immune simulations and molecular docking predicted its ability to promote the development of B-cell and memory T-helper cells, IgG, and the binding to both TLR2 and TLR4.

Despite the improvements to control strategies and their implementation, *Ascaris* remains the most prevalent STH worldwide^1^. Currently, there is no vaccine developed against *A. lumbricoides* or *A. suum* for use in either humans or pigs^11,28^. Recently, helminth vaccine research has included the development of subunit vaccines^11,87^. The polypeptide developed here using an in silico approach included epitopes from previously tested *Ascaris* antigens and proteins recently identified as potential vaccine candidates using bioinformatics methodologies^12,14,15,18,43^. This antigen selection differed from previous studies that chose proteins based only on a bioinformatics approach or known vaccine targets^15,88^. The selected proteins were present in both *A. lumbricoides* and *A. suum*, due to the known potential for host cross-infection and hybridization between both species^8,89^. Having a vaccine that targets both species is increasingly relevant in areas where both humans and pigs co-exist^3,7,90^. This contrasts to the approach used in previous in silico designed vaccines that focused on antigens only present in *A. lumbricoides* proteome^88^. The epitope selection in the present study also differs from the previous ones. Both B-cell and HTC epitopes were selected while the chimeric vaccine was created solely based on B-cell epitopes^15^. Nonetheless, there is overlap between our selected B-cell epitopes for As14 and As37 and the ones in the aforementioned study. There was no overlap between As16 selected epitopes between the studies that could be explained by the different program version used (Bepipred 1.0 and Bepipred 2.0). The HTC epitope selection in the present study was based on binding to a 27 MHC-II human reference allele set, contrasting to the two-allele set used by the previous in silico study^88^, and were present in extracellular domains of the selected antigens. MHC-II molecules appear to have a prominent role in the control of nematode infections in mammals by presenting antigenic peptides (through their epitopes) to HTC^11,91^. This makes the discovery of epitopes that bind to these molecules a priority for the design of multi-epitope/subunit-based vaccines. The role of B-cells in *Ascaris* infection is still not fully understood but it is known to reduce larvae burden through antibody production^15,17,23,92^. The selection of non-allergic, non-toxic, and proteins and epitopes without orthologues in humans or pigs should minimise the risk of adverse reactions to the vaccine. It is important to note that the selected B-cell epitopes were also predicted to only induce IgG production. Even though IgG is known to be a major component in most *Ascaris* vaccination assays^12,13,15,17,32^, both IgE and IgA also play a role in disease control^12,17,24,33^. For this reason, exploring other epitopes could be an interesting proposal for further optimizing a multi-epitope *Ascaris* vaccine.

The development of a subunit vaccine requires the selection of appropriate linkers between the selected epitopes as epitopes tend to not promote an immunological response by themselves^54^. These linkers not only improve antigen processing and presentation, they should also provide the needed structural rigidity and flexibility to the vaccine^54,93^. The selection of EAAAK and GSGSG epitope linkers was made based on previous nematode subunit vaccines studies^15,41,42^. The EAAAK linker was used to fuse the vaccine with the RS09 TLR-4 agonist. TLR-4 mediates immune responses to *Ascaris* and the use of a synthetic agonist is known to be a safe alternative to natural agonists^24,30,55,94^. The use of other adjuvants, such as Monophosphoryl lipid A (MPLA™), as already been proven useful in controlling *Ascaris* infection and should be considered in future assays^15,95^. The high antigenicity, non-allergenicity and non-toxicity scores strengthen the potential usefulness as a good vaccination candidate. The multi-epitope vaccine was predicted to be both stable (with a half-life of 10 h) and soluble upon expression *E. coli*, supporting its potential for production in these commonly used expression bacteria systems. This was also reinforced by the ability to optimize the peptide coding sequence for expression in *E. coli* k12 using the pET30a (+) vector. *E. coli* is the most popular system for the production of recombinant proteins and has been optimized for its fast growth and high protein production output^96^.

Knowledge of vaccine secondary and tertiary structure is a staple in understanding their potential usefulness^97^. The 2D structure analysis indicated an existence of 55.1% coils and 30.6% α-sheets, allowing the protein to be recognized by antibodies^97^. This structure is similar to what was predicted for other in silico vaccine studies^40,42,95^. The predicted protein tertiary structure improved after refinement. The Ramachandran plot highlighted the presence of 98% of the residues in favoured and allowed regions, indicating the high-quality of the produced model^72^. Although analysis by ERRAT and PROVE were negative, the analysis with ProSA-Web, VERIFY 3D and PROCHECK validate the protein 3D model. These results goes in line to what was found in other in silico vaccine design studies, where residues in disallowed regions using Ramachandran plot were between zero and five per cent^40–42,98^. A high-quality 3D model of a polypeptide is needed to assess the presence of discontinuous B-cell epitopes and to accurately predict its binding to proteins responsible for the hosts immune response. Even though the designed vaccine was constructed with linear B-cell epitopes, these are only responsible for 10% of the B-cell response^76^. With the prediction of eight discontinuous B-cell epitopes, it is foreseen to be able to stimulate B-cell binding and recognition. Alongside B-cells, both TLR2 and TLR4 are known to promote immune response against *Ascaris* and play a vital role in innate immune responses^24,30^. The docking study with Cluspro 2.0 was able to predict binding between the designed vaccine and both TLR2 and TLR4. Even though the polypeptide binding was higher to TLR4 than to TLR2, this could be explained due to the addition of the synthetic TLR4 agonist to the N-terminal of the peptide.

The immune simulation scenarios suggested the development of a favourable immune response after immunization. This immune simulation server delivers the vaccine through injection. It has been shown that it be worth considering other delivery methods as they could be more effective^19^. Despite a similar increase in both B-cell and HTC populations in both scenarios, antibody titer was higher in the three-dose scenario than the two-dose scenario, suggesting that it could be more useful to prevent further larvae infection^24^. The predicted increase in IgG production is a desirable scenario for ascariasis as it is associated with larvae reduction and protection against infection^15,17^. IL-4 and IL-10 stimulation was observed in both scenarios, with the three-dose scenario having a smaller peak after the second booster. Nevertheless, the differences observed might not prove to be enough to justify a second booster based on economic or immunological considerations. The immune simulation reinforces the epitope analysis data that these would induce the production of IL-4, IL-10 and IgG. These results are similar to what was found in previous *Ascaris* vaccination assays, including the most recent ones that used a multi-epitope vaccine^14,15,17,20,32^.

Alongside with other recently published vaccines, the one developed in this study should be ready to be expressed and studied both in vitro and in vivo using animal models^15,88^. These studies should be able to provide a clear understanding of the vaccine capacity to stimulate an adequate immune response and if this could be improved by a new combination of epitopes or antigens.

## Conclusion

In this study we have proposed a multi-epitope polypeptide, based on a variety of in silico analyses that jointly investigate important immunological properties that such a vaccine should include. This strategy will allow rapid prediction of the potential of vaccines before they are tested experimentally. The developed vaccine uses B-cell and HTC epitopes from proteins existent in both *A. lumbricoides* and *A. suum* and is predicted to be able to develop protection against these zoonotic species. The present study provides an alternative development approach to vaccines against *Ascaris* that could prove useful in future studies.

## Supplementary Information

Supplementary Information.

## Data Availability

The datasets used and analysed in this study are available in the article/Supplementary information. For further information do contact the corresponding author.
