# Supplementary material for: In silico design of a polypeptide as a vaccine candidate against ascariasis
Source: Sci Rep. 2023 Mar 2;13:3504. doi: 10.1038/s41598-023-30445-x (PMC9981566; doi:10.1038/s41598-023-30445-x)
Supplement: Supplementary file 1 — Supplementary Information. [file 41598_2023_30445_MOESM1_ESM.docx]

*In silico* design of a polypeptide as a vaccine candidate against ascariasis.

Francisco M. D. Evangelista^1^, Arnoud H. M. van Vliet^1^, Scott P. Lawton^2^, Martha Betson^1*^

^1^ School of Veterinary Medicine, Faculty of Health and Medical Sciences, University of Surrey, Guildford GU2 7AL, United Kingdom

^2^ Centre for Epidemiology and Planetary Health, Department of Veterinary and Animal Sciences, Northern Faculty, Scotland’s Rural University College (SRUC), An Lòchran, Inverness, IV2 5NA, United Kingdom

*** Corresponding author**:

Martha Betson

Email: [m.betson@surrey.ac.uk](mailto:m.betson@surrey.ac.uk)

Supplementary Table S1 - Results of IL4pred and IL-10Pred for each selected HTC epitope.

| [**HTC**](https://webs.iiitd.edu.in/raghava/il4pred/multi_submitfreq_S.php?ran=67544) **Epitope** | [**IL-4**](https://webs.iiitd.edu.in/raghava/il4pred/multi_submitfreq_S.php?ran=67544) **Pred score** | **IL-4** [**Prediction**](https://webs.iiitd.edu.in/raghava/il4pred/multi_submitfreq_S.php?ran=67544) | **IL-10 Pred score** | **IL-10 Prediction** |
| --- | --- | --- | --- | --- |
| [**LRLLRALRPLRVINR**](https://webs.iiitd.edu.in/raghava/il4pred/pepsearch_S.php?seq=LRLLRALRPLRVINR&thval=0.2) | 0.28 | IL-4 inducer | 0.22 | Not IL-10 inducer |
| [**FKNFGMAFLTLFRIA**](https://webs.iiitd.edu.in/raghava/il4pred/pepsearch_S.php?seq=FKNFGMAFLTLFRIA&thval=0.2) | 1.22 | IL-4 inducer | 0.25 | Not IL-10 inducer |
| [**HTFRRFITAISLLDR**](https://webs.iiitd.edu.in/raghava/il4pred/pepsearch_S.php?seq=HTFRRFITAISLLDR&thval=0.2) | 0.20 | Not IL-4 inducer | 0.49 | IL-10 inducer |
| [**NQEGVVHILSRKIFD**](https://webs.iiitd.edu.in/raghava/il4pred/pepsearch_S.php?seq=NQEGVVHILSRKIFD&thval=0.2) | 0.17 | Not IL-4 inducer | 0.41 | IL-10 inducer |
| [**NCLKYFANFFFYRFG**](https://webs.iiitd.edu.in/raghava/il4pred/pepsearch_S.php?seq=NCLKYFANFFFYRFG&thval=0.2) | 0.34 | IL-4 inducer | 0.76 | IL-10 inducer |
| [**SLFLRPMRVALALLN**](https://webs.iiitd.edu.in/raghava/il4pred/pepsearch_S.php?seq=SLFLRPMRVALALLN&thval=0.2) | 0.19 | Not IL-4 inducer | 0.33 | IL-10 inducer |
| [**NNNFHTFPAAILVLF**](https://webs.iiitd.edu.in/raghava/il4pred/pepsearch_S.php?seq=NNNFHTFPAAILVLF&thval=0.2) | 0.19 | Not IL-4 inducer | 0.01 | Not IL-10 inducer |
| [**ERSLLCLTLSNPLRK**](https://webs.iiitd.edu.in/raghava/il4pred/pepsearch_S.php?seq=ERSLLCLTLSNPLRK&thval=0.2) | -0.16 | Not IL-4 inducer | 0.89 | IL-10 inducer |
| [**TTELKQDNRFSFRLD**](https://webs.iiitd.edu.in/raghava/il4pred/pepsearch_S.php?seq=TTELKQDNRFSFRLD&thval=0.2) | 1.11 | IL-4 inducer | 1.10 | IL-10 inducer |
| [**VMVMEFRAKSILKPT**](https://webs.iiitd.edu.in/raghava/il4pred/pepsearch_S.php?seq=VMVMEFRAKSILKPT&thval=0.2) | 0.25 | IL-4 inducer | 0.33 | IL-10 inducer |
| [**AEYEKAHAAAIAKFS**](https://webs.iiitd.edu.in/raghava/il4pred/pepsearch_S.php?seq=AEYEKAHAAAIAKFS&thval=0.2) | 0.43 | IL-4 inducer | 0.72 | IL-10 inducer |
| [**YTNKFKAFKAELKAH**](https://webs.iiitd.edu.in/raghava/il4pred/pepsearch_S.php?seq=YTNKFKAFKAELKAH&thval=0.2) | 1.27 | IL-4 inducer | 0.22 | Not IL-10 inducer |
| [**KITSLLQSLPAAVQA**](https://webs.iiitd.edu.in/raghava/il4pred/pepsearch_S.php?seq=KITSLLQSLPAAVQA&thval=0.2) | 0.01 | Not IL-4 inducer | 0.42 | IL-10 inducer |
| [**KVLIIFVAIVVIAFA**](https://webs.iiitd.edu.in/raghava/il4pred/pepsearch_S.php?seq=KVLIIFVAIVVIAFA&thval=0.2) | -0.65 | Not IL-4 inducer | 0.13 | Not IL-10 inducer |

Supplementary Data S1 – Amino acid sequence of the multi-epitope vaccine

APPHALSEAAAKLRLLRALRPLRVINRGSGSGFKNFGMAFLTLFRIAGSGSGHTFRRFITAISLLDRGSGSGNQEGVVHILSRKIFDGSGSGNCLKYFANFFFYRFGGSGSGSLFLRPMRVALALLNGSGSGNNNFHTFPAAILVLFGSGSGERSLLCLTLSNPLRKGSGSGTTELKQDNRFSFRLDGSGSGVMVMEFRAKSILKPTGSGSGAEYEKAHAAAIAKFSGSGSGYTNKFKAFKAELKAHGSGSGKITSLLQSLPAAVQAGSGSGKVLIIFVAIVVIAFAGSGSGDATGVDMQPVENYNGSGSGSIPPKSVERGSGSGLSQSDHHILPRFANFVDDRTESLRSVTIQLLCSLRGSGSGRQQFTLTFPYFSDGKFKGSGSGLLSVHLKNDDDSIESTGSGSGVDPSFDPVIPKEEVIGSGSGSNEEDRGPVYNAGSGSGALNDETHIHRNNNGSGSGPQGAPTFTRKPQILQKTSDSGDGSGSGPLDDGADDAGSGSGEGQTPSRVPPFGSGSGEDAKLNGIQKRQKIKETMEGSGSGTQMQQGKARAEAAGSGSGDNPNLKGREKQQKITSL

Supplementary Data S2 – Optimized DNA sequence of the multi-epitope vaccine

GCTCCGCCGCACGCTCTGTCTGAAGCTGCTGCTAAACTGCGTCTGCTGCGTGCTCTGCGTCCGCTGCGTGTTATCAACCGTGGTTCTGGTTCTGGTTTCAAAAACTTCGGTATGGCTTTCCTGACCCTGTTCCGTATCGCTGGTTCTGGTTCTGGTCACACCTTCCGTCGTTTCATCACCGCTATCTCTCTGCTGGACCGTGGTTCTGGTTCTGGTAACCAGGAAGGTGTTGTTCACATCCTGTCTCGTAAAATCTTCGACGGTTCTGGTTCTGGTAACTGCCTGAAATACTTCGCTAACTTCTTCTTCTACCGTTTCGGTGGTTCTGGTTCTGGTTCTCTGTTCCTGCGTCCGATGCGTGTTGCTCTGGCTCTGCTGAACGGTTCTGGTTCTGGTAACAACAACTTCCACACCTTCCCGGCTGCTATCCTGGTTCTGTTCGGTTCTGGTTCTGGTGAACGTTCTCTGCTGTGCCTGACCCTGTCTAACCCGCTGCGTAAAGGTTCTGGTTCTGGTACCACCGAACTGAAACAGGACAACCGTTTCTCTTTCCGTCTGGACGGTTCTGGTTCTGGTGTTATGGTTATGGAATTCCGTGCTAAATCTATCCTGAAACCGACCGGTTCTGGTTCTGGTGCTGAATACGAAAAAGCTCACGCTGCTGCTATCGCTAAATTCTCTGGTTCTGGTTCTGGTTACACCAACAAATTCAAAGCTTTCAAAGCTGAACTGAAAGCTCACGGTTCTGGTTCTGGTAAAATCACCTCTCTGCTGCAGTCTCTGCCGGCTGCTGTTCAGGCTGGTTCTGGTTCTGGTAAAGTTCTGATCATCTTCGTTGCTATCGTTGTTATCGCTTTCGCTGGTTCTGGTTCTGGTGACGCTACCGGTGTTGACATGCAGCCGGTTGAAAACTACAACGGTTCTGGTTCTGGTTCTATCCCGCCGAAATCTGTTGAACGTGGTTCTGGTTCTGGTCTGTCTCAGTCTGACCACCACATCCTGCCGCGTTTCGCTAACTTCGTTGACGACCGTACCGAATCTCTGCGTTCTGTTACCATCCAGCTGCTGTGCTCTCTGCGTGGTTCTGGTTCTGGTCGTCAGCAGTTCACCCTGACCTTCCCGTACTTCTCTGACGGTAAATTCAAAGGTTCTGGTTCTGGTCTGCTGTCTGTTCACCTGAAAAACGACGACGACTCTATCGAATCTACCGGTTCTGGTTCTGGTGTTGACCCGTCTTTCGACCCGGTTATCCCGAAAGAAGAAGTTATCGGTTCTGGTTCTGGTTCTAACGAAGAAGACCGTGGTCCGGTTTACAACGCTGGTTCTGGTTCTGGTGCTCTGAACGACGAAACCCACATCCACCGTAACAACAACGGTTCTGGTTCTGGTCCGCAGGGTGCTCCGACCTTCACCCGTAAACCGCAGATCCTGCAGAAAACCTCTGACTCTGGTGACGGTTCTGGTTCTGGTCCGCTGGACGACGGTGCTGACGACGCTGGTTCTGGTTCTGGTGAAGGTCAGACCCCGTCTCGTGTTCCGCCGTTCGGTTCTGGTTCTGGTGAAGACGCTAAACTGAACGGTATCCAGAAACGTCAGAAAATCAAAGAAACCATGGAAGGTTCTGGTTCTGGTACCCAGATGCAGCAGGGTAAAGCTCGTGCTGAAGCTGCTGGTTCTGGTTCTGGTGACAACCCGAACCTGAAAGGTCGTGAAAAACAGCAGAAAATCACCTCTCTG

Supplementary Data S3 – Optimized DNA sequence of the multi-epitope vaccine as used in SnapGene tool.

ATGCATATGGCTCCGCCGCACGCTCTGTCTGAAGCTGCTGCTAAACTGCGTCTGCTGCGTGCTCTGCGTCCGCTGCGTGTTATCAACCGTGGTTCTGGTTCTGGTTTCAAAAACTTCGGTATGGCTTTCCTGACCCTGTTCCGTATCGCTGGTTCTGGTTCTGGTCACACCTTCCGTCGTTTCATCACCGCTATCTCTCTGCTGGACCGTGGTTCTGGTTCTGGTAACCAGGAAGGTGTTGTTCACATCCTGTCTCGTAAAATCTTCGACGGTTCTGGTTCTGGTAACTGCCTGAAATACTTCGCTAACTTCTTCTTCTACCGTTTCGGTGGTTCTGGTTCTGGTTCTCTGTTCCTGCGTCCGATGCGTGTTGCTCTGGCTCTGCTGAACGGTTCTGGTTCTGGTAACAACAACTTCCACACCTTCCCGGCTGCTATCCTGGTTCTGTTCGGTTCTGGTTCTGGTGAACGTTCTCTGCTGTGCCTGACCCTGTCTAACCCGCTGCGTAAAGGTTCTGGTTCTGGTACCACCGAACTGAAACAGGACAACCGTTTCTCTTTCCGTCTGGACGGTTCTGGTTCTGGTGTTATGGTTATGGAATTCCGTGCTAAATCTATCCTGAAACCGACCGGTTCTGGTTCTGGTGCTGAATACGAAAAAGCTCACGCTGCTGCTATCGCTAAATTCTCTGGTTCTGGTTCTGGTTACACCAACAAATTCAAAGCTTTCAAAGCTGAACTGAAAGCTCACGGTTCTGGTTCTGGTAAAATCACCTCTCTGCTGCAGTCTCTGCCGGCTGCTGTTCAGGCTGGTTCTGGTTCTGGTAAAGTTCTGATCATCTTCGTTGCTATCGTTGTTATCGCTTTCGCTGGTTCTGGTTCTGGTGACGCTACCGGTGTTGACATGCAGCCGGTTGAAAACTACAACGGTTCTGGTTCTGGTTCTATCCCGCCGAAATCTGTTGAACGTGGTTCTGGTTCTGGTCTGTCTCAGTCTGACCACCACATCCTGCCGCGTTTCGCTAACTTCGTTGACGACCGTACCGAATCTCTGCGTTCTGTTACCATCCAGCTGCTGTGCTCTCTGCGTGGTTCTGGTTCTGGTCGTCAGCAGTTCACCCTGACCTTCCCGTACTTCTCTGACGGTAAATTCAAAGGTTCTGGTTCTGGTCTGCTGTCTGTTCACCTGAAAAACGACGACGACTCTATCGAATCTACCGGTTCTGGTTCTGGTGTTGACCCGTCTTTCGACCCGGTTATCCCGAAAGAAGAAGTTATCGGTTCTGGTTCTGGTTCTAACGAAGAAGACCGTGGTCCGGTTTACAACGCTGGTTCTGGTTCTGGTGCTCTGAACGACGAAACCCACATCCACCGTAACAACAACGGTTCTGGTTCTGGTCCGCAGGGTGCTCCGACCTTCACCCGTAAACCGCAGATCCTGCAGAAAACCTCTGACTCTGGTGACGGTTCTGGTTCTGGTCCGCTGGACGACGGTGCTGACGACGCTGGTTCTGGTTCTGGTGAAGGTCAGACCCCGTCTCGTGTTCCGCCGTTCGGTTCTGGTTCTGGTGAAGACGCTAAACTGAACGGTATCCAGAAACGTCAGAAAATCAAAGAAACCATGGAAGGTTCTGGTTCTGGTACCCAGATGCAGCAGGGTAAAGCTCGTGCTGAAGCTGCTGGTTCTGGTTCTGGTGACAACCCGAACCTGAAAGGTCGTGAAAAACAGCAGAAAATCACCTCTCTGCTCGAGCATCACCATCACCATCACTAA

Supplementary Table S2 - Discontinuous B-cell epitopes of the multi-epitope vaccine as identified with ElliPro.

| **Epitopes** | **Residues (amino acids)** | **Number of residues** | **Score** |
| --- | --- | --- | --- |
| 1 | A:S481, A:D482, A:S483, A:G484, A:D485, A:G486, A:S487, A:G488, A:S489, A:G490, A:P491, A:L492, A:G495, A:A496, A:D497, A:D498, A:A499, A:G500, A:S501, A:G502, A:S503, A:G504, A:E505, A:G506, A:Q507, A:T508, A:K535, A:E536, A:T537, A:M538, A:E539, A:G540, A:S541, A:G542, A:S543, A:G544, A:T545, A:Q546, A:M547, A:Q548, A:Q549, A:G550, A:K551, A:A552, A:R553, A:A554, A:E555, A:A556, A:A557, A:G558, A:S559, A:G560, A:S561, A:G562, A:D563, A:N564, A:P565, A:N566, A:L567, A:K568, A:G569, A:R570, A:E571, A:K572, A:Q573, A:Q574, A:K575, A:I576, A:T577, A:S578, A:L579 | 71 | 0.782 |
| 2 | A:P164, A:R166, A:G168, A:S169, A:G170, A:S171, A:G172, A:T173, A:T174, A:E175, A:L176 | 11 | 0.721 |
| 3 | A:A1, A:P2, A:P3, A:H4, A:A5, A:L6, A:S7, A:E8, A:A9, A:A10, A:A11, A:K12, A:L13, A:R14, A:L15, A:L16, A:R17, A:A18, A:L19, A:R20, A:P21, A:L22, A:V24, A:I25, A:N26, A:R27, A:G28, A:S29, A:G30, A:S31, A:G32, A:F33, A:K34, A:N35, A:F36, A:L41, A:T42, A:L43, A:F44, A:R45, A:I46, A:A47, A:G48, A:S49, A:G50, A:S51, A:G52, A:H53, A:T54, A:F55, A:R56, A:R57, A:F58, A:I59, A:T60, A:A61, A:I62, A:S63, A:L64, A:L65, A:D66, A:R67, A:G68, A:S69, A:G70, A:S71, A:G72, A:N73, A:Q74, A:E75, A:G76, A:V77, A:V78, A:I85, A:F86, A:D87, A:G88, A:S89, A:G90, A:S91, A:G92, A:N93, A:C94, A:L95, A:K96, A:Y97, A:F98, A:A99, A:N100, A:F103, A:F106, A:G107, A:G108, A:S109, A:G110, A:S111, A:G112, A:S113, A:L114, A:R117, A:F147, A:G148, A:S149, A:G150, A:S151, A:G152, A:E153, A:R154, A:D187, A:G188, A:S189, A:G190, A:S191, A:G192, A:V193 | 115 | 0.718 |
| 4 | A:R344, A:S347, A:L348, A:G383, A:S384, A:G385, A:I417, A:P418, A:K419, A:E420, A:E421, A:V422, A:I423, A:G424, A:S425, A:G426, A:S427, A:G428, A:S429, A:N430, A:E431, A:E432, A:D433, A:R434, A:P436, A:Y438, A:L447, A:N448, A:D449, A:E450, A:T451, A:H452, A:I453, A:H454, A:R455, A:N456, A:N457, A:N458, A:G459, A:S460, A:G461, A:S462, A:G463, A:P464, A:Q465, A:G466, A:A467, A:P468, A:T469 | 49 | 0.69 |
| 5 | A:N127, A:G128, A:S129, A:G130, A:S131, A:G132, A:N133, A:N134, A:N135, A:F136, A:F139, A:L165, A:K167 | 13 | 0.65 |
| 6 | A:L204, A:K205, A:P206, A:T207, A:G208, A:S209, A:G210, A:S211, A:G212, A:A213, A:E214, A:Y215, A:Q266, A:A267, A:G268, A:S269, A:G270, A:S271, A:G272, A:K273, A:L359, A:R360, A:G361, A:S362, A:G363, A:S364, A:G365, A:R366, A:Q368, A:K394, A:N395, A:D396, A:D397, A:D398, A:S399, A:I400, A:E401, A:S402 | 38 | 0.642 |
| 7 | A:E346, A:D378, A:G379, A:K380, A:K382, A:G516, A:S517, A:G518, A:S519, A:G520, A:E521, A:D522, A:K524, A:L525, A:N526, A:Q529 | 16 | 0.519 |
| 8 | A:N304, A:N306, A:G307, A:S308, A:G309, A:S310, A:G311 | 7 | 0.518 |

Supplementary Table S3 – PRODIGY assessment of molecular docking between the vaccine and TLR4 and TLR2.

| **TLR4** | | | | **TLR2** | | | |
| --- | --- | --- | --- | --- | --- | --- | --- |
| Binding affinity ΔG (kcal mol^-1^) | Dissociation constant Kd (M) | Number of contacts | Global energy | Binding affinity ΔG (kcal mol^-1^) | Dissociation constant Kd (M) | Number of contacts | Global energy |
| -19.00 | 4.20E-14 | 177.00 | -108.83 | -12.60 | 1.30E-09 | 108.00 | -161.86 |

Supplementary Table S4 – Hydrogen bonds predicted by Ligplot+ in the molecular docking model between the vaccine and TLR2. Chain A corresponds to TLR2 and Chain B corresponds to the designed polypeptide.

| **Number of contacts** | **Atom number** | **Atom name** | **Residue name** | **Residue number** | **Chain** | **Atom number** | **Atom name** | **Residue name** | **Residue number** | **Chain** | **Atom number** |
| --- | --- | --- | --- | --- | --- | --- | --- | --- | --- | --- | --- |
| 1 | 146 | NH2 | ARG | 20 | B | 161 | OD1 | ASN | 379 | A | 2.816 |
| 2 | 145 | NH1 | ARG | 20 | B | 161 | OD1 | ASN | 379 | A | 2.664 |
| 3 | 54 | NH2 | ARG | 14 | B | 43 | OH | TYR | 376 | A | 2.623 |
| 4 | 51 | NE | ARG | 14 | B | 43 | OH | TYR | 376 | A | 2.812 |
| 5 | 43 | OH | TYR | 376 | A | 75 | O | LEU | 13 | B | 2.745 |
| 6 | 53 | NH1 | ARG | 14 | B | 122 | OE1 | GLU | 375 | A | 2.734 |
| 7 | 20 | NH2 | ARG | 83 | B | 154 | OD2 | ASP | 327 | A | 2.740 |
| 8 | 19 | NH1 | ARG | 83 | B | 154 | OD2 | ASP | 327 | A | 2.758 |
| 9 | 9 | NH2 | ARG | 45 | B | 135 | OH | TYR | 326 | A | 2.797 |
| 10 | 8 | NH1 | ARG | 45 | B | 135 | OH | TYR | 326 | A | 2.694 |
| 11 | 23 | N | PHE | 325 | A | 224 | O | SER | 82 | B | 3.061 |
| 12 | 93 | OH | TYR | 323 | A | 11 | O | ARG | 45 | B | 2.762 |
| 13 | 1 | N | ARG | 45 | B | 93 | OH | TYR | 323 | A | 2.887 |
| 14 | 65 | NH2 | ARG | 296 | A | 276 | O | ALA | 142 | B | 2.666 |
| 15 | 65 | NH2 | ARG | 296 | A | 185 | O | ALA | 141 | B | 2.655 |
| 16 | 64 | NH1 | ARG | 296 | A | 189 | OG1 | THR | 160 | B | 2.707 |
| 17 | 76 | N | ASN | 294 | A | 22 | O | ARG | 83 | B | 2.801 |
| 18 | 246 | OG1 | THR | 138 | B | 256 | O | VAL | 292 | A | 2.970 |
| 19 | 103 | NH1 | ARG | 166 | B | 171 | OE2 | GLU | 264 | A | 2.908 |
| 20 | 103 | NH1 | ARG | 166 | B | 113 | NE2 | HIS | 238 | A | 2.901 |

Supplementary Table S5 – Non-bonded contacts predicted by Ligplot+ in the molecular docking model between the vaccine and TLR2. Chain A corresponds to TLR2 and Chain B corresponds to the designed polypeptide.

| **Number of contacts** | **Atom number** | **Atom name** | **Residue name** | **Residue number** | **Chain** | **Atom number** | **Atom name** | **Residue name** | **Residue number** | **Chain** | **Atom number** |
| --- | --- | --- | --- | --- | --- | --- | --- | --- | --- | --- | --- |
| 1 | 323 | CD2 | LEU | 6 | B | 315 | CE1 | HIS | 398 | A | 3.748 |
| 2 | 385 | CB | ALA | 9 | B | 314 | NE2 | HIS | 398 | A | 3.872 |
| 3 | 323 | CD2 | LEU | 6 | B | 312 | ND1 | HIS | 398 | A | 3.727 |
| 4 | 144 | CZ | ARG | 20 | B | 161 | OD1 | ASN | 379 | A | 3.163 |
| 5 | 146 | NH2 | ARG | 20 | B | 160 | CG | ASN | 379 | A | 3.638 |
| 6 | 145 | NH1 | ARG | 20 | B | 160 | CG | ASN | 379 | A | 3.568 |
| 7 | 146 | NH2 | ARG | 20 | B | 159 | CB | ASN | 379 | A | 3.838 |
| 8 | 52 | CZ | ARG | 14 | B | 43 | OH | TYR | 376 | A | 3.061 |
| 9 | 47 | CA | ARG | 14 | B | 43 | OH | TYR | 376 | A | 3.737 |
| 10 | 74 | C | LEU | 13 | B | 43 | OH | TYR | 376 | A | 3.364 |
| 11 | 54 | NH2 | ARG | 14 | B | 42 | CZ | TYR | 376 | A | 3.107 |
| 12 | 52 | CZ | ARG | 14 | B | 42 | CZ | TYR | 376 | A | 3.870 |
| 13 | 75 | O | LEU | 13 | B | 42 | CZ | TYR | 376 | A | 3.144 |
| 14 | 54 | NH2 | ARG | 14 | B | 41 | CE2 | TYR | 376 | A | 3.654 |
| 15 | 72 | CD1 | LEU | 13 | B | 41 | CE2 | TYR | 376 | A | 3.320 |
| 16 | 72 | CD1 | LEU | 13 | B | 40 | CD2 | TYR | 376 | A | 3.725 |
| 17 | 213 | NE | ARG | 17 | B | 39 | CE1 | TYR | 376 | A | 3.836 |
| 18 | 54 | NH2 | ARG | 14 | B | 39 | CE1 | TYR | 376 | A | 3.821 |
| 19 | 75 | O | LEU | 13 | B | 39 | CE1 | TYR | 376 | A | 3.134 |
| 20 | 214 | CZ | ARG | 17 | B | 123 | OE2 | GLU | 375 | A | 3.589 |
| 21 | 52 | CZ | ARG | 14 | B | 123 | OE2 | GLU | 375 | A | 3.541 |
| 22 | 52 | CZ | ARG | 14 | B | 122 | OE1 | GLU | 375 | A | 3.200 |
| 23 | 216 | NH2 | ARG | 17 | B | 121 | CD | GLU | 375 | A | 2.985 |
| 24 | 54 | NH2 | ARG | 14 | B | 121 | CD | GLU | 375 | A | 2.957 |
| 25 | 53 | NH1 | ARG | 14 | B | 121 | CD | GLU | 375 | A | 3.583 |
| 26 | 52 | CZ | ARG | 14 | B | 121 | CD | GLU | 375 | A | 3.670 |
| 27 | 72 | CD1 | LEU | 13 | B | 417 | CG2 | VAL | 373 | A | 3.767 |
| 28 | 73 | CD2 | LEU | 13 | B | 372 | O | LEU | 371 | A | 3.849 |
| 29 | 72 | CD1 | LEU | 13 | B | 372 | O | LEU | 371 | A | 3.405 |
| 30 | 146 | NH2 | ARG | 20 | B | 362 | CD2 | LEU | 350 | A | 3.870 |
| 31 | 143 | NE | ARG | 20 | B | 362 | CD2 | LEU | 350 | A | 3.764 |
| 32 | 72 | CD1 | LEU | 13 | B | 207 | O | PHE | 349 | A | 3.823 |
| 33 | 289 | CD1 | LEU | 19 | B | 205 | CZ | PHE | 349 | A | 3.778 |
| 34 | 289 | CD1 | LEU | 19 | B | 204 | CE2 | PHE | 349 | A | 3.580 |
| 35 | 70 | CB | LEU | 13 | B | 202 | CD2 | PHE | 349 | A | 3.816 |
| 36 | 70 | CB | LEU | 13 | B | 200 | CG | PHE | 349 | A | 3.877 |
| 37 | 73 | CD2 | LEU | 13 | B | 199 | CB | PHE | 349 | A | 3.575 |
| 38 | 72 | CD1 | LEU | 13 | B | 199 | CB | PHE | 349 | A | 3.723 |
| 39 | 71 | CG | LEU | 13 | B | 199 | CB | PHE | 349 | A | 3.834 |
| 40 | 70 | CB | LEU | 13 | B | 199 | CB | PHE | 349 | A | 3.646 |
| 41 | 18 | CZ | ARG | 83 | B | 154 | OD2 | ASP | 327 | A | 3.167 |
| 42 | 281 | CD1 | LEU | 43 | B | 154 | OD2 | ASP | 327 | A | 3.635 |
| 43 | 20 | NH2 | ARG | 83 | B | 152 | CG | ASP | 327 | A | 3.850 |
| 44 | 19 | NH1 | ARG | 83 | B | 152 | CG | ASP | 327 | A | 3.406 |
| 45 | 19 | NH1 | ARG | 83 | B | 151 | CB | ASP | 327 | A | 3.200 |
| 46 | 7 | CZ | ARG | 45 | B | 135 | OH | TYR | 326 | A | 3.172 |
| 47 | 9 | NH2 | ARG | 45 | B | 134 | CZ | TYR | 326 | A | 3.271 |
| 48 | 8 | NH1 | ARG | 45 | B | 134 | CZ | TYR | 326 | A | 3.362 |
| 49 | 7 | CZ | ARG | 45 | B | 134 | CZ | TYR | 326 | A | 3.729 |
| 50 | 8 | NH1 | ARG | 45 | B | 133 | CE2 | TYR | 326 | A | 3.554 |
| 51 | 9 | NH2 | ARG | 45 | B | 131 | CE1 | TYR | 326 | A | 3.617 |
| 52 | 279 | CB | LEU | 43 | B | 31 | CZ | PHE | 325 | A | 3.675 |
| 53 | 290 | CD2 | LEU | 19 | B | 31 | CZ | PHE | 325 | A | 3.831 |
| 54 | 20 | NH2 | ARG | 83 | B | 30 | CE2 | PHE | 325 | A | 3.308 |
| 55 | 18 | CZ | ARG | 83 | B | 30 | CE2 | PHE | 325 | A | 3.771 |
| 56 | 279 | CB | LEU | 43 | B | 30 | CE2 | PHE | 325 | A | 3.655 |
| 57 | 6 | NE | ARG | 45 | B | 29 | CE1 | PHE | 325 | A | 3.853 |
| 58 | 4 | CG | ARG | 45 | B | 29 | CE1 | PHE | 325 | A | 3.877 |
| 59 | 290 | CD2 | LEU | 19 | B | 29 | CE1 | PHE | 325 | A | 3.740 |
| 60 | 20 | NH2 | ARG | 83 | B | 28 | CD2 | PHE | 325 | A | 3.538 |
| 61 | 18 | CZ | ARG | 83 | B | 28 | CD2 | PHE | 325 | A | 3.565 |
| 62 | 17 | NE | ARG | 83 | B | 28 | CD2 | PHE | 325 | A | 3.674 |
| 63 | 15 | CG | ARG | 83 | B | 28 | CD2 | PHE | 325 | A | 3.892 |
| 64 | 394 | O | THR | 42 | B | 28 | CD2 | PHE | 325 | A | 3.899 |
| 65 | 284 | O | LEU | 43 | B | 27 | CD1 | PHE | 325 | A | 3.883 |
| 66 | 284 | O | LEU | 43 | B | 26 | CG | PHE | 325 | A | 3.875 |
| 67 | 15 | CG | ARG | 83 | B | 25 | CB | PHE | 325 | A | 3.676 |
| 68 | 12 | N | ARG | 83 | B | 25 | CB | PHE | 325 | A | 3.771 |
| 69 | 224 | O | SER | 82 | B | 25 | CB | PHE | 325 | A | 3.223 |
| 70 | 223 | C | SER | 82 | B | 25 | CB | PHE | 325 | A | 3.415 |
| 71 | 221 | CB | SER | 82 | B | 25 | CB | PHE | 325 | A | 3.609 |
| 72 | 224 | O | SER | 82 | B | 24 | CA | PHE | 325 | A | 3.685 |
| 73 | 335 | N | ILE | 85 | B | 410 | CD2 | LEU | 324 | A | 3.650 |
| 74 | 340 | CD1 | ILE | 85 | B | 95 | O | TYR | 323 | A | 3.224 |
| 75 | 10 | C | ARG | 45 | B | 93 | OH | TYR | 323 | A | 3.394 |
| 76 | 4 | CG | ARG | 45 | B | 93 | OH | TYR | 323 | A | 3.874 |
| 77 | 3 | CB | ARG | 45 | B | 93 | OH | TYR | 323 | A | 3.201 |
| 78 | 2 | CA | ARG | 45 | B | 93 | OH | TYR | 323 | A | 3.280 |
| 79 | 11 | O | ARG | 45 | B | 92 | CZ | TYR | 323 | A | 3.669 |
| 80 | 3 | CB | ARG | 45 | B | 92 | CZ | TYR | 323 | A | 3.747 |
| 81 | 11 | O | ARG | 45 | B | 91 | CE2 | TYR | 323 | A | 3.711 |
| 82 | 344 | CA | SER | 89 | B | 240 | CZ | PHE | 322 | A | 3.686 |
| 83 | 267 | O | GLY | 88 | B | 240 | CZ | PHE | 322 | A | 3.265 |
| 84 | 267 | O | GLY | 88 | B | 239 | CE2 | PHE | 322 | A | 3.085 |
| 85 | 344 | CA | SER | 89 | B | 238 | CE1 | PHE | 322 | A | 3.766 |
| 86 | 267 | O | GLY | 88 | B | 238 | CE1 | PHE | 322 | A | 3.604 |
| 87 | 267 | O | GLY | 88 | B | 237 | CD2 | PHE | 322 | A | 3.276 |
| 88 | 267 | O | GLY | 88 | B | 236 | CD1 | PHE | 322 | A | 3.764 |
| 89 | 267 | O | GLY | 88 | B | 235 | CG | PHE | 322 | A | 3.611 |
| 90 | 8 | NH1 | ARG | 45 | B | 354 | CD1 | ILE | 319 | A | 3.417 |
| 91 | 8 | NH1 | ARG | 45 | B | 352 | CG2 | ILE | 319 | A | 3.495 |
| 92 | 190 | CG2 | THR | 160 | B | 65 | NH2 | ARG | 296 | A | 3.339 |
| 93 | 296 | CG2 | ILE | 143 | B | 65 | NH2 | ARG | 296 | A | 3.806 |
| 94 | 275 | C | ALA | 142 | B | 65 | NH2 | ARG | 296 | A | 3.371 |
| 95 | 184 | C | ALA | 141 | B | 65 | NH2 | ARG | 296 | A | 3.532 |
| 96 | 190 | CG2 | THR | 160 | B | 64 | NH1 | ARG | 296 | A | 3.410 |
| 97 | 188 | CB | THR | 160 | B | 64 | NH1 | ARG | 296 | A | 3.617 |
| 98 | 190 | CG2 | THR | 160 | B | 63 | CZ | ARG | 296 | A | 3.528 |
| 99 | 189 | OG1 | THR | 160 | B | 63 | CZ | ARG | 296 | A | 3.530 |
| 100 | 276 | O | ALA | 142 | B | 63 | CZ | ARG | 296 | A | 3.884 |
| 101 | 185 | O | ALA | 141 | B | 63 | CZ | ARG | 296 | A | 3.746 |
| 102 | 183 | CB | ALA | 141 | B | 83 | O | ASN | 294 | A | 3.799 |
| 103 | 297 | CG1 | ILE | 143 | B | 81 | ND2 | ASN | 294 | A | 3.873 |
| 104 | 298 | CD1 | ILE | 143 | B | 80 | OD1 | ASN | 294 | A | 3.413 |
| 105 | 297 | CG1 | ILE | 143 | B | 80 | OD1 | ASN | 294 | A | 3.488 |
| 106 | 330 | CD | LYS | 84 | B | 80 | OD1 | ASN | 294 | A | 3.410 |
| 107 | 329 | CG | LYS | 84 | B | 80 | OD1 | ASN | 294 | A | 3.586 |
| 108 | 185 | O | ALA | 141 | B | 79 | CG | ASN | 294 | A | 3.166 |
| 109 | 185 | O | ALA | 141 | B | 78 | CB | ASN | 294 | A | 3.469 |
| 110 | 181 | N | ALA | 141 | B | 78 | CB | ASN | 294 | A | 3.518 |
| 111 | 179 | C | PRO | 140 | B | 78 | CB | ASN | 294 | A | 3.616 |
| 112 | 177 | CB | PRO | 140 | B | 78 | CB | ASN | 294 | A | 3.707 |
| 113 | 176 | CA | PRO | 140 | B | 78 | CB | ASN | 294 | A | 3.502 |
| 114 | 22 | O | ARG | 83 | B | 77 | CA | ASN | 294 | A | 3.691 |
| 115 | 21 | C | ARG | 83 | B | 76 | N | ASN | 294 | A | 3.876 |
| 116 | 14 | CB | ARG | 83 | B | 76 | N | ASN | 294 | A | 3.843 |
| 117 | 178 | CG | PRO | 140 | B | 196 | O | GLY | 293 | A | 3.854 |
| 118 | 177 | CB | PRO | 140 | B | 196 | O | GLY | 293 | A | 3.899 |
| 119 | 176 | CA | PRO | 140 | B | 196 | O | GLY | 293 | A | 3.105 |
| 120 | 175 | CD | PRO | 140 | B | 196 | O | GLY | 293 | A | 3.887 |
| 121 | 178 | CG | PRO | 140 | B | 195 | C | GLY | 293 | A | 3.801 |
| 122 | 176 | CA | PRO | 140 | B | 195 | C | GLY | 293 | A | 3.694 |
| 123 | 22 | O | ARG | 83 | B | 195 | C | GLY | 293 | A | 3.313 |
| 124 | 178 | CG | PRO | 140 | B | 194 | CA | GLY | 293 | A | 3.745 |
| 125 | 22 | O | ARG | 83 | B | 194 | CA | GLY | 293 | A | 3.199 |
| 126 | 248 | C | THR | 138 | B | 256 | O | VAL | 292 | A | 3.864 |
| 127 | 245 | CB | THR | 138 | B | 256 | O | VAL | 292 | A | 3.461 |
| 128 | 245 | CB | THR | 138 | B | 252 | CB | VAL | 292 | A | 3.618 |
| 129 | 102 | CZ | ARG | 166 | B | 171 | OE2 | GLU | 264 | A | 3.253 |
| 130 | 102 | CZ | ARG | 166 | B | 170 | OE1 | GLU | 264 | A | 3.878 |
| 131 | 104 | NH2 | ARG | 166 | B | 169 | CD | GLU | 264 | A | 3.115 |
| 132 | 304 | OG1 | THR | 174 | B | 114 | CE1 | HIS | 238 | A | 3.337 |
| 133 | 103 | NH1 | ARG | 166 | B | 114 | CE1 | HIS | 238 | A | 3.695 |
| 134 | 304 | OG1 | THR | 174 | B | 112 | CD2 | HIS | 238 | A | 3.283 |
| 135 | 263 | O | THR | 173 | B | 112 | CD2 | HIS | 238 | A | 3.415 |
| 136 | 262 | C | THR | 173 | B | 112 | CD2 | HIS | 238 | A | 3.810 |
| 137 | 271 | O | GLY | 172 | B | 112 | CD2 | HIS | 238 | A | 3.092 |
| 138 | 304 | OG1 | THR | 174 | B | 110 | CG | HIS | 238 | A | 3.194 |
| 139 | 271 | O | GLY | 172 | B | 110 | CG | HIS | 238 | A | 3.415 |
| 140 | 304 | OG1 | THR | 174 | B | 109 | CB | HIS | 238 | A | 3.898 |
| 141 | 271 | O | GLY | 172 | B | 109 | CB | HIS | 238 | A | 3.315 |
| 142 | 258 | CA | THR | 173 | B | 231 | O | THR | 236 | A | 3.608 |
| 143 | 270 | C | GLY | 172 | B | 231 | O | THR | 236 | A | 3.105 |
| 144 | 269 | CA | GLY | 172 | B | 231 | O | THR | 236 | A | 3.678 |
| 145 | 261 | CG2 | THR | 173 | B | 229 | CG2 | THR | 236 | A | 3.825 |
| 146 | 401 | NE2 | HIS | 137 | B | 229 | CG2 | THR | 236 | A | 3.796 |
| 147 | 261 | CG2 | THR | 173 | B | 228 | OG1 | THR | 236 | A | 3.696 |
| 148 | 261 | CG2 | THR | 173 | B | 227 | CB | THR | 236 | A | 3.616 |
| 149 | 258 | CA | THR | 173 | B | 227 | CB | THR | 236 | A | 3.898 |
| 150 | 269 | CA | GLY | 172 | B | 379 | NE2 | HIS | 210 | A | 3.204 |

Supplementary Table S6 – Salt bridges predicted by Ligplot+ in the molecular docking model between the vaccine and TLR2. Chain A corresponds to TLR2 and Chain B corresponds to the designed polypeptide.

| **Number of contacts** | **Atom number** | **Atom name** | **Residue name** | **Residue number** | **Chain** | **Atom number** | **Atom name** | **Residue name** | **Residue number** | **Chain** | **Atom number** |
| --- | --- | --- | --- | --- | --- | --- | --- | --- | --- | --- | --- |
| 1 | 123 | OE2 | GLU | 375 | A | 216 | NH2 | ARG | 17 | B | 2.790 |
| 2 | 170 | OE1 | GLU | 264 | A | 104 | NH2 | ARG | 166 | B | 2.706 |

Supplementary Table S7 – Hydrogen bonds predicted by Ligplot+ in the molecular docking model between the vaccine and TLR4. Chain A corresponds to TLR4 and Chain B corresponds to the designed polypeptide.

| **Number of contacts** | **Atom number** | **Atom name** | **Residue name** | **Residue number** | **Chain** | **Atom number** | **Atom name** | **Residue name** | **Residue number** | **Chain** | **Atom number** |
| --- | --- | --- | --- | --- | --- | --- | --- | --- | --- | --- | --- |
| 1 | 392 | ND2 | ASN | 127 | B | 401 | O | VAL | 602 | A | 2.730 |
| 2 | 126 | ND2 | ASN | 133 | B | 438 | O | SER | 570 | A | 2.812 |
| 3 | 6 | NE | ARG | 120 | B | 149 | OD1 | ASP | 550 | A | 3.015 |
| 4 | 233 | NE2 | GLN | 547 | A | 125 | OD1 | ASN | 133 | B | 2.875 |
| 5 | 92 | NH2 | ARG | 117 | B | 258 | OG | SER | 504 | A | 2.663 |
| 6 | 89 | NE | ARG | 117 | B | 258 | OG | SER | 504 | A | 3.321 |
| 7 | 345 | NZ | LYS | 477 | A | 212 | O | LEU | 95 | B | 2.709 |
| 8 | 286 | OG | SER | 91 | B | 335 | OE1 | GLU | 474 | A | 2.931 |
| 9 | 19 | NH1 | ARG | 57 | B | 218 | OD2 | ASP | 428 | A | 2.728 |
| 10 | 518 | N | GLY | 52 | B | 107 | OE2 | GLU | 425 | A | 2.962 |
| 11 | 156 | OG | SER | 51 | B | 106 | OE1 | GLU | 425 | A | 2.877 |
| 12 | 17 | NE | ARG | 57 | B | 141 | OD1 | ASP | 405 | A | 2.902 |
| 13 | 64 | NH2 | ARG | 14 | B | 201 | OE1 | GLU | 287 | A | 2.733 |
| 14 | 63 | NH1 | ARG | 14 | B | 201 | OE1 | GLU | 287 | A | 2.691 |
| 15 | 115 | NE | ARG | 27 | B | 279 | OD1 | ASN | 265 | A | 2.924 |
| 16 | 39 | NE | ARG | 17 | B | 133 | OD1 | ASP | 209 | A | 3.172 |
| 17 | 41 | NH1 | ARG | 17 | B | 252 | OD2 | ASP | 181 | A | 2.764 |
| 18 | 30 | NH1 | ARG | 20 | B | 272 | OE2 | GLU | 178 | A | 2.747 |
| 19 | 318 | NZ | LYS | 130 | A | 362 | O | PRO | 21 | B | 2.723 |
| 20 | 75 | NH2 | ARG | 23 | B | 224 | OG | SER | 86 | A | 2.715 |
| 21 | 74 | NH1 | ARG | 23 | B | 224 | OG | SER | 86 | A | 2.713 |
| 22 | 75 | NH2 | ARG | 23 | B | 353 | OD2 | ASP | 84 | A | 2.548 |
| 23 | 184 | NZ | LYS | 34 | B | 193 | OE2 | GLU | 42 | A | 2.521 |
| 24 | 184 | NZ | LYS | 34 | B | 192 | OE1 | GLU | 42 | A | 2.590 |
| 25 | 303 | N | GLU | 31 | A | 98 | OG | SER | 249 | B | 2.902 |
| 26 | 95 | N | SER | 249 | B | 243 | OE1 | GLU | 27 | A | 2.903 |

Supplementary Table S8 – Non-contact bonds predicted by Ligplot+ in the molecular docking model between the vaccine and TLR4. Chain A corresponds to TLR4 and Chain B corresponds to the designed polypeptide.

| **Number of contacts** | **Atom number** | **Atom name** | **Residue name** | **Residue number** | **Chain** | **Atom number** | **Atom name** | **Residue name** | **Residue number** | **Chain** | **Atom number** |
| --- | --- | --- | --- | --- | --- | --- | --- | --- | --- | --- | --- |
| 1 | 4 | CG | ARG | 120 | B | 515 | NH2 | ARG | 606 | A | 3.780 |
| 2 | 610 | SD | MET | 119 | B | 515 | NH2 | ARG | 606 | A | 3.698 |
| 3 | 526 | CD1 | LEU | 123 | B | 514 | NH1 | ARG | 606 | A | 3.647 |
| 4 | 610 | SD | MET | 119 | B | 513 | CZ | ARG | 606 | A | 3.784 |
| 5 | 526 | CD1 | LEU | 123 | B | 384 | OE2 | GLU | 603 | A | 3.449 |
| 6 | 524 | CB | LEU | 123 | B | 384 | OE2 | GLU | 603 | A | 3.713 |
| 7 | 4 | CG | ARG | 120 | B | 384 | OE2 | GLU | 603 | A | 3.772 |
| 8 | 3 | CB | ARG | 120 | B | 384 | OE2 | GLU | 603 | A | 3.683 |
| 9 | 2 | CA | ARG | 120 | B | 384 | OE2 | GLU | 603 | A | 3.401 |
| 10 | 4 | CG | ARG | 120 | B | 383 | OE1 | GLU | 603 | A | 3.891 |
| 11 | 390 | CG | ASN | 127 | B | 401 | O | VAL | 602 | A | 3.870 |
| 12 | 80 | CB | SER | 129 | B | 804 | O | LEU | 601 | A | 3.842 |
| 13 | 80 | CB | SER | 129 | B | 796 | O | LEU | 600 | A | 3.891 |
| 14 | 80 | CB | SER | 129 | B | 506 | O | GLN | 599 | A | 3.127 |
| 15 | 79 | CA | SER | 129 | B | 506 | O | GLN | 599 | A | 3.743 |
| 16 | 623 | CE | LYS | 167 | B | 501 | OE1 | GLN | 599 | A | 3.638 |
| 17 | 622 | CD | LYS | 167 | B | 501 | OE1 | GLN | 599 | A | 3.819 |
| 18 | 5 | CD | ARG | 120 | B | 587 | ND2 | ASN | 575 | A | 3.279 |
| 19 | 4 | CG | ARG | 120 | B | 587 | ND2 | ASN | 575 | A | 3.785 |
| 20 | 3 | CB | ARG | 120 | B | 587 | ND2 | ASN | 575 | A | 3.397 |
| 21 | 83 | O | SER | 129 | B | 53 | CZ | PHE | 573 | A | 3.296 |
| 22 | 78 | N | SER | 129 | B | 53 | CZ | PHE | 573 | A | 3.664 |
| 23 | 367 | O | ALA | 124 | B | 53 | CZ | PHE | 573 | A | 3.444 |
| 24 | 366 | C | ALA | 124 | B | 53 | CZ | PHE | 573 | A | 3.882 |
| 25 | 83 | O | SER | 129 | B | 52 | CE2 | PHE | 573 | A | 3.318 |
| 26 | 82 | C | SER | 129 | B | 52 | CE2 | PHE | 573 | A | 3.895 |
| 27 | 80 | CB | SER | 129 | B | 52 | CE2 | PHE | 573 | A | 3.717 |
| 28 | 79 | CA | SER | 129 | B | 52 | CE2 | PHE | 573 | A | 3.717 |
| 29 | 78 | N | SER | 129 | B | 52 | CE2 | PHE | 573 | A | 3.202 |
| 30 | 367 | O | ALA | 124 | B | 52 | CE2 | PHE | 573 | A | 3.079 |
| 31 | 366 | C | ALA | 124 | B | 52 | CE2 | PHE | 573 | A | 3.532 |
| 32 | 365 | CB | ALA | 124 | B | 52 | CE2 | PHE | 573 | A | 3.683 |
| 33 | 413 | OD1 | ASN | 134 | B | 51 | CE1 | PHE | 573 | A | 3.601 |
| 34 | 412 | CG | ASN | 134 | B | 51 | CE1 | PHE | 573 | A | 3.429 |
| 35 | 411 | CB | ASN | 134 | B | 51 | CE1 | PHE | 573 | A | 3.366 |
| 36 | 83 | O | SER | 129 | B | 51 | CE1 | PHE | 573 | A | 3.553 |
| 37 | 83 | O | SER | 129 | B | 50 | CD2 | PHE | 573 | A | 3.608 |
| 38 | 80 | CB | SER | 129 | B | 50 | CD2 | PHE | 573 | A | 3.729 |
| 39 | 365 | CB | ALA | 124 | B | 50 | CD2 | PHE | 573 | A | 3.851 |
| 40 | 83 | O | SER | 129 | B | 49 | CD1 | PHE | 573 | A | 3.825 |
| 41 | 83 | O | SER | 129 | B | 48 | CG | PHE | 573 | A | 3.863 |
| 42 | 615 | CA | GLY | 130 | B | 265 | O | ALA | 572 | A | 3.704 |
| 43 | 82 | C | SER | 129 | B | 265 | O | ALA | 572 | A | 3.045 |
| 44 | 80 | CB | SER | 129 | B | 265 | O | ALA | 572 | A | 3.725 |
| 45 | 83 | O | SER | 129 | B | 264 | C | ALA | 572 | A | 3.750 |
| 46 | 126 | ND2 | ASN | 133 | B | 263 | CB | ALA | 572 | A | 3.708 |
| 47 | 125 | OD1 | ASN | 133 | B | 263 | CB | ALA | 572 | A | 3.593 |
| 48 | 124 | CG | ASN | 133 | B | 263 | CB | ALA | 572 | A | 3.534 |
| 49 | 615 | CA | GLY | 130 | B | 263 | CB | ALA | 572 | A | 3.731 |
| 50 | 83 | O | SER | 129 | B | 263 | CB | ALA | 572 | A | 3.765 |
| 51 | 126 | ND2 | ASN | 133 | B | 437 | C | SER | 570 | A | 3.816 |
| 52 | 8 | NH1 | ARG | 120 | B | 684 | CD1 | LEU | 553 | A | 3.424 |
| 53 | 723 | CB | LEU | 116 | B | 684 | CD1 | LEU | 553 | A | 3.887 |
| 54 | 7 | CZ | ARG | 120 | B | 493 | OG | SER | 552 | A | 2.742 |
| 55 | 5 | CD | ARG | 120 | B | 493 | OG | SER | 552 | A | 3.409 |
| 56 | 6 | NE | ARG | 120 | B | 492 | CB | SER | 552 | A | 3.758 |
| 57 | 5 | CD | ARG | 120 | B | 492 | CB | SER | 552 | A | 3.773 |
| 58 | 7 | CZ | ARG | 120 | B | 150 | OD2 | ASP | 550 | A | 3.598 |
| 59 | 7 | CZ | ARG | 120 | B | 149 | OD1 | ASP | 550 | A | 3.325 |
| 60 | 9 | NH2 | ARG | 120 | B | 148 | CG | ASP | 550 | A | 3.002 |
| 61 | 7 | CZ | ARG | 120 | B | 148 | CG | ASP | 550 | A | 3.815 |
| 62 | 6 | NE | ARG | 120 | B | 148 | CG | ASP | 550 | A | 3.781 |
| 63 | 124 | CG | ASN | 133 | B | 233 | NE2 | GLN | 547 | A | 3.811 |
| 64 | 414 | ND2 | ASN | 134 | B | 231 | CD | GLN | 547 | A | 3.814 |
| 65 | 125 | OD1 | ASN | 133 | B | 231 | CD | GLN | 547 | A | 3.792 |
| 66 | 125 | OD1 | ASN | 133 | B | 230 | CG | GLN | 547 | A | 3.744 |
| 67 | 414 | ND2 | ASN | 134 | B | 229 | CB | GLN | 547 | A | 3.883 |
| 68 | 7 | CZ | ARG | 120 | B | 677 | OG | SER | 528 | A | 3.471 |
| 69 | 9 | NH2 | ARG | 120 | B | 676 | CB | SER | 528 | A | 3.276 |
| 70 | 88 | CD | ARG | 117 | B | 786 | ND2 | ASN | 526 | A | 3.693 |
| 71 | 90 | CZ | ARG | 117 | B | 484 | OE1 | GLN | 505 | A | 3.446 |
| 72 | 718 | CD2 | LEU | 114 | B | 484 | OE1 | GLN | 505 | A | 3.291 |
| 73 | 92 | NH2 | ARG | 117 | B | 483 | CD | GLN | 505 | A | 3.019 |
| 74 | 92 | NH2 | ARG | 117 | B | 482 | CG | GLN | 505 | A | 3.824 |
| 75 | 90 | CZ | ARG | 117 | B | 258 | OG | SER | 504 | A | 3.430 |
| 76 | 92 | NH2 | ARG | 117 | B | 257 | CB | SER | 504 | A | 3.865 |
| 77 | 298 | CD | LYS | 96 | B | 472 | CD1 | PHE | 500 | A | 3.618 |
| 78 | 299 | CE | LYS | 96 | B | 471 | CG | PHE | 500 | A | 3.662 |
| 79 | 298 | CD | LYS | 96 | B | 471 | CG | PHE | 500 | A | 3.808 |
| 80 | 299 | CE | LYS | 96 | B | 470 | CB | PHE | 500 | A | 3.580 |
| 81 | 92 | NH2 | ARG | 117 | B | 778 | CB | ALA | 479 | A | 3.676 |
| 82 | 211 | C | LEU | 95 | B | 345 | NZ | LYS | 477 | A | 3.754 |
| 83 | 212 | O | LEU | 95 | B | 344 | CE | LYS | 477 | A | 3.451 |
| 84 | 300 | NZ | LYS | 96 | B | 430 | CG2 | VAL | 475 | A | 3.822 |
| 85 | 299 | CE | LYS | 96 | B | 430 | CG2 | VAL | 475 | A | 3.632 |
| 86 | 288 | O | SER | 91 | B | 430 | CG2 | VAL | 475 | A | 3.302 |
| 87 | 287 | C | SER | 91 | B | 430 | CG2 | VAL | 475 | A | 3.673 |
| 88 | 299 | CE | LYS | 96 | B | 429 | CG1 | VAL | 475 | A | 3.749 |
| 89 | 285 | CB | SER | 91 | B | 335 | OE1 | GLU | 474 | A | 3.179 |
| 90 | 284 | CA | SER | 91 | B | 335 | OE1 | GLU | 474 | A | 3.730 |
| 91 | 207 | CB | LEU | 95 | B | 579 | OH | TYR | 451 | A | 3.745 |
| 92 | 207 | CB | LEU | 95 | B | 577 | CE2 | TYR | 451 | A | 3.621 |
| 93 | 710 | CA | GLY | 92 | B | 576 | CD2 | TYR | 451 | A | 3.738 |
| 94 | 455 | CZ | PHE | 103 | B | 566 | NE2 | HIS | 431 | A | 3.407 |
| 95 | 453 | CE1 | PHE | 103 | B | 566 | NE2 | HIS | 431 | A | 3.842 |
| 96 | 455 | CZ | PHE | 103 | B | 565 | CD2 | HIS | 431 | A | 3.833 |
| 97 | 18 | CZ | ARG | 57 | B | 218 | OD2 | ASP | 428 | A | 3.318 |
| 98 | 18 | CZ | ARG | 57 | B | 217 | OD1 | ASP | 428 | A | 3.880 |
| 99 | 20 | NH2 | ARG | 57 | B | 216 | CG | ASP | 428 | A | 2.830 |
| 100 | 19 | NH1 | ARG | 57 | B | 216 | CG | ASP | 428 | A | 3.380 |
| 101 | 18 | CZ | ARG | 57 | B | 216 | CG | ASP | 428 | A | 3.519 |
| 102 | 20 | NH2 | ARG | 57 | B | 215 | CB | ASP | 428 | A | 3.267 |
| 103 | 19 | NH1 | ARG | 57 | B | 557 | CE1 | HIS | 426 | A | 3.837 |
| 104 | 209 | CD1 | LEU | 95 | B | 556 | NE2 | HIS | 426 | A | 3.270 |
| 105 | 209 | CD1 | LEU | 95 | B | 555 | CD2 | HIS | 426 | A | 3.749 |
| 106 | 157 | C | SER | 51 | B | 107 | OE2 | GLU | 425 | A | 3.660 |
| 107 | 155 | CB | SER | 51 | B | 107 | OE2 | GLU | 425 | A | 3.358 |
| 108 | 154 | CA | SER | 51 | B | 107 | OE2 | GLU | 425 | A | 3.346 |
| 109 | 155 | CB | SER | 51 | B | 106 | OE1 | GLU | 425 | A | 3.236 |
| 110 | 154 | CA | SER | 51 | B | 106 | OE1 | GLU | 425 | A | 3.597 |
| 111 | 156 | OG | SER | 51 | B | 105 | CD | GLU | 425 | A | 3.710 |
| 112 | 155 | CB | SER | 51 | B | 105 | CD | GLU | 425 | A | 3.629 |
| 113 | 154 | CA | SER | 51 | B | 105 | CD | GLU | 425 | A | 3.894 |
| 114 | 455 | CZ | PHE | 103 | B | 773 | CZ | PHE | 408 | A | 3.481 |
| 115 | 18 | CZ | ARG | 57 | B | 142 | OD2 | ASP | 405 | A | 3.855 |
| 116 | 18 | CZ | ARG | 57 | B | 141 | OD1 | ASP | 405 | A | 3.217 |
| 117 | 20 | NH2 | ARG | 57 | B | 140 | CG | ASP | 405 | A | 2.967 |
| 118 | 18 | CZ | ARG | 57 | B | 140 | CG | ASP | 405 | A | 3.680 |
| 119 | 17 | NE | ARG | 57 | B | 140 | CG | ASP | 405 | A | 3.673 |
| 120 | 16 | CD | ARG | 57 | B | 547 | OH | TYR | 403 | A | 3.139 |
| 121 | 15 | CG | ARG | 57 | B | 547 | OH | TYR | 403 | A | 3.299 |
| 122 | 14 | CB | ARG | 57 | B | 547 | OH | TYR | 403 | A | 3.851 |
| 123 | 600 | NE | ARG | 56 | B | 669 | CE1 | PHE | 377 | A | 3.428 |
| 124 | 599 | CD | ARG | 56 | B | 669 | CE1 | PHE | 377 | A | 3.743 |
| 125 | 706 | CG2 | THR | 60 | B | 762 | CG2 | THR | 357 | A | 3.799 |
| 126 | 698 | CD1 | LEU | 16 | B | 754 | NH1 | ARG | 289 | A | 3.567 |
| 127 | 62 | CZ | ARG | 14 | B | 201 | OE1 | GLU | 287 | A | 3.073 |
| 128 | 64 | NH2 | ARG | 14 | B | 200 | CD | GLU | 287 | A | 3.675 |
| 129 | 63 | NH1 | ARG | 14 | B | 200 | CD | GLU | 287 | A | 3.877 |
| 130 | 405 | CB | PRO | 3 | B | 423 | OE2 | GLU | 286 | A | 3.416 |
| 131 | 404 | CA | PRO | 3 | B | 423 | OE2 | GLU | 286 | A | 3.856 |
| 132 | 406 | CG | PRO | 3 | B | 422 | OE1 | GLU | 286 | A | 3.517 |
| 133 | 405 | CB | PRO | 3 | B | 422 | OE1 | GLU | 286 | A | 3.348 |
| 134 | 405 | CB | PRO | 3 | B | 421 | CD | GLU | 286 | A | 3.780 |
| 135 | 116 | CZ | ARG | 27 | B | 326 | OE1 | GLU | 266 | A | 3.763 |
| 136 | 118 | NH2 | ARG | 27 | B | 325 | CD | GLU | 266 | A | 2.973 |
| 137 | 116 | CZ | ARG | 27 | B | 279 | OD1 | ASN | 265 | A | 3.726 |
| 138 | 114 | CD | ARG | 27 | B | 279 | OD1 | ASN | 265 | A | 3.859 |
| 139 | 112 | CB | ARG | 27 | B | 279 | OD1 | ASN | 265 | A | 3.638 |
| 140 | 115 | NE | ARG | 27 | B | 278 | CG | ASN | 265 | A | 3.877 |
| 141 | 115 | NE | ARG | 27 | B | 741 | CD2 | PHE | 263 | A | 3.672 |
| 142 | 63 | NH1 | ARG | 14 | B | 656 | CD | ARG | 257 | A | 3.596 |
| 143 | 63 | NH1 | ARG | 14 | B | 654 | CB | ARG | 257 | A | 3.293 |
| 144 | 62 | CZ | ARG | 14 | B | 464 | NE2 | HIS | 256 | A | 3.736 |
| 145 | 592 | CB | ALA | 11 | B | 464 | NE2 | HIS | 256 | A | 3.482 |
| 146 | 63 | NH1 | ARG | 14 | B | 463 | CD2 | HIS | 256 | A | 3.090 |
| 147 | 690 | CB | SER | 7 | B | 462 | ND1 | HIS | 256 | A | 3.769 |
| 148 | 60 | CD | ARG | 14 | B | 175 | CE1 | HIS | 229 | A | 3.766 |
| 149 | 59 | CG | ARG | 14 | B | 175 | CE1 | HIS | 229 | A | 3.813 |
| 150 | 58 | CB | ARG | 14 | B | 175 | CE1 | HIS | 229 | A | 3.325 |
| 151 | 293 | O | ALA | 10 | B | 175 | CE1 | HIS | 229 | A | 3.315 |
| 152 | 292 | C | ALA | 10 | B | 175 | CE1 | HIS | 229 | A | 3.732 |
| 153 | 60 | CD | ARG | 14 | B | 174 | NE2 | HIS | 229 | A | 3.781 |
| 154 | 58 | CB | ARG | 14 | B | 174 | NE2 | HIS | 229 | A | 3.850 |
| 155 | 591 | CA | ALA | 11 | B | 174 | NE2 | HIS | 229 | A | 3.620 |
| 156 | 292 | C | ALA | 10 | B | 174 | NE2 | HIS | 229 | A | 3.351 |
| 157 | 291 | CB | ALA | 10 | B | 174 | NE2 | HIS | 229 | A | 3.688 |
| 158 | 291 | CB | ALA | 10 | B | 173 | CD2 | HIS | 229 | A | 3.663 |
| 159 | 40 | CZ | ARG | 17 | B | 133 | OD1 | ASP | 209 | A | 3.442 |
| 160 | 42 | NH2 | ARG | 17 | B | 132 | CG | ASP | 209 | A | 3.025 |
| 161 | 40 | CZ | ARG | 17 | B | 132 | CG | ASP | 209 | A | 3.819 |
| 162 | 39 | NE | ARG | 17 | B | 132 | CG | ASP | 209 | A | 3.805 |
| 163 | 42 | NH2 | ARG | 17 | B | 131 | CB | ASP | 209 | A | 3.216 |
| 164 | 291 | CB | ALA | 10 | B | 649 | ND2 | ASN | 205 | A | 3.799 |
| 165 | 291 | CB | ALA | 10 | B | 648 | OD1 | ASN | 205 | A | 3.533 |
| 166 | 40 | CZ | ARG | 17 | B | 252 | OD2 | ASP | 181 | A | 3.233 |
| 167 | 42 | NH2 | ARG | 17 | B | 250 | CG | ASP | 181 | A | 2.907 |
| 168 | 41 | NH1 | ARG | 17 | B | 250 | CG | ASP | 181 | A | 3.488 |
| 169 | 40 | CZ | ARG | 17 | B | 250 | CG | ASP | 181 | A | 3.607 |
| 170 | 42 | NH2 | ARG | 17 | B | 249 | CB | ASP | 181 | A | 3.775 |
| 171 | 28 | NE | ARG | 20 | B | 375 | CE1 | HIS | 179 | A | 3.626 |
| 172 | 27 | CD | ARG | 20 | B | 375 | CE1 | HIS | 179 | A | 3.709 |
| 173 | 29 | CZ | ARG | 20 | B | 374 | NE2 | HIS | 179 | A | 3.630 |
| 174 | 31 | NH2 | ARG | 20 | B | 373 | CD2 | HIS | 179 | A | 3.866 |
| 175 | 29 | CZ | ARG | 20 | B | 373 | CD2 | HIS | 179 | A | 3.601 |
| 176 | 28 | NE | ARG | 20 | B | 373 | CD2 | HIS | 179 | A | 3.716 |
| 177 | 29 | CZ | ARG | 20 | B | 272 | OE2 | GLU | 178 | A | 3.330 |
| 178 | 31 | NH2 | ARG | 20 | B | 270 | CD | GLU | 178 | A | 3.100 |
| 179 | 30 | NH1 | ARG | 20 | B | 270 | CD | GLU | 178 | A | 3.589 |
| 180 | 29 | CZ | ARG | 20 | B | 270 | CD | GLU | 178 | A | 3.791 |
| 181 | 27 | CD | ARG | 20 | B | 165 | OE2 | GLU | 154 | A | 3.710 |
| 182 | 27 | CD | ARG | 20 | B | 164 | OE1 | GLU | 154 | A | 3.497 |
| 183 | 26 | CG | ARG | 20 | B | 164 | OE1 | GLU | 154 | A | 3.698 |
| 184 | 25 | CB | ARG | 20 | B | 164 | OE1 | GLU | 154 | A | 3.394 |
| 185 | 30 | NH1 | ARG | 20 | B | 163 | CD | GLU | 154 | A | 3.792 |
| 186 | 27 | CD | ARG | 20 | B | 163 | CD | GLU | 154 | A | 3.506 |
| 187 | 30 | NH1 | ARG | 20 | B | 162 | CG | GLU | 154 | A | 3.343 |
| 188 | 361 | C | PRO | 21 | B | 318 | NZ | LYS | 130 | A | 3.819 |
| 189 | 362 | O | PRO | 21 | B | 317 | CE | LYS | 130 | A | 3.148 |
| 190 | 74 | NH1 | ARG | 23 | B | 731 | CB | THR | 110 | A | 3.544 |
| 191 | 75 | NH2 | ARG | 23 | B | 533 | CG2 | ILE | 108 | A | 3.577 |
| 192 | 74 | NH1 | ARG | 23 | B | 533 | CG2 | ILE | 108 | A | 3.597 |
| 193 | 73 | CZ | ARG | 23 | B | 533 | CG2 | ILE | 108 | A | 3.804 |
| 194 | 73 | CZ | ARG | 23 | B | 224 | OG | SER | 86 | A | 3.133 |
| 195 | 75 | NH2 | ARG | 23 | B | 223 | CB | SER | 86 | A | 3.567 |
| 196 | 74 | NH1 | ARG | 23 | B | 223 | CB | SER | 86 | A | 3.651 |
| 197 | 73 | CZ | ARG | 23 | B | 353 | OD2 | ASP | 84 | A | 3.717 |
| 198 | 75 | NH2 | ARG | 23 | B | 351 | CG | ASP | 84 | A | 3.045 |
| 199 | 183 | CE | LYS | 34 | B | 193 | OE2 | GLU | 42 | A | 3.857 |
| 200 | 183 | CE | LYS | 34 | B | 192 | OE1 | GLU | 42 | A | 3.842 |
| 201 | 184 | NZ | LYS | 34 | B | 191 | CD | GLU | 42 | A | 2.906 |
| 202 | 98 | OG | SER | 249 | B | 305 | CB | GLU | 31 | A | 3.578 |
| 203 | 98 | OG | SER | 249 | B | 304 | CA | GLU | 31 | A | 3.781 |
| 204 | 98 | OG | SER | 249 | B | 642 | C | VAL | 30 | A | 3.613 |
| 205 | 98 | OG | SER | 249 | B | 638 | CA | VAL | 30 | A | 3.585 |
| 206 | 97 | CB | SER | 249 | B | 636 | O | CYS | 29 | A | 3.267 |
| 207 | 96 | CA | SER | 249 | B | 636 | O | CYS | 29 | A | 3.349 |
| 208 | 99 | C | SER | 249 | B | 246 | O | GLU | 27 | A | 3.112 |
| 209 | 96 | CA | SER | 249 | B | 246 | O | GLU | 27 | A | 3.354 |
| 210 | 96 | CA | SER | 249 | B | 243 | OE1 | GLU | 27 | A | 3.764 |
| 211 | 629 | C | GLY | 248 | B | 243 | OE1 | GLU | 27 | A | 3.660 |
| 212 | 628 | CA | GLY | 248 | B | 243 | OE1 | GLU | 27 | A | 3.469 |

Supplementary Table S9 – Salt bridges predicted by Ligplot+ in the molecular docking model between the vaccine and TLR4. Chain A corresponds to TLR4 and Chain B corresponds to the designed polypeptide.

| **Number of contacts** | **Atom number** | **Atom name** | **Residue name** | **Residue number** | **Chain** | **Atom number** | **Atom name** | **Residue name** | **Residue number** | **Chain** | **Atom number** |
| --- | --- | --- | --- | --- | --- | --- | --- | --- | --- | --- | --- |
| 1 | 149 | OD1 | ASP | 550 | A | 9 | NH2 | ARG | 120 | B | 2.769 |
| 2 | 444 | OD2 | ASP | 502 | A | 89 | NE | ARG | 117 | B | 3.861 |
| 3 | 141 | OD1 | ASP | 405 | A | 20 | NH2 | ARG | 57 | B | 2.732 |
| 4 | 327 | OE2 | GLU | 266 | A | 118 | NH2 | ARG | 27 | B | 2.627 |
| 5 | 133 | OD1 | ASP | 209 | A | 42 | NH2 | ARG | 17 | B | 2.824 |
| 6 | 164 | OE1 | GLU | 154 | A | 30 | NH1 | ARG | 20 | B | 3.891 |
